# Supplementary material for: Synthesis of Anti-Inflammatory Spirostene-Pyrazole Conjugates by a Consecutive Multicomponent Reaction of Diosgenin with Oxalyl Chloride, Arylalkynes and Hydrazines or Hydrazones
Source: Molecules. 2021 Dec 28;27(1):162. doi: 10.3390/molecules27010162 (PMC8746855; doi:10.3390/molecules27010162)

Compound 2 ((22*R*,25*R*)-spirost-5-en-3*β*-yl 2-chloro-2-oxoacetate), ^{1}H NMR, 400 MHz, $CDCl_3$

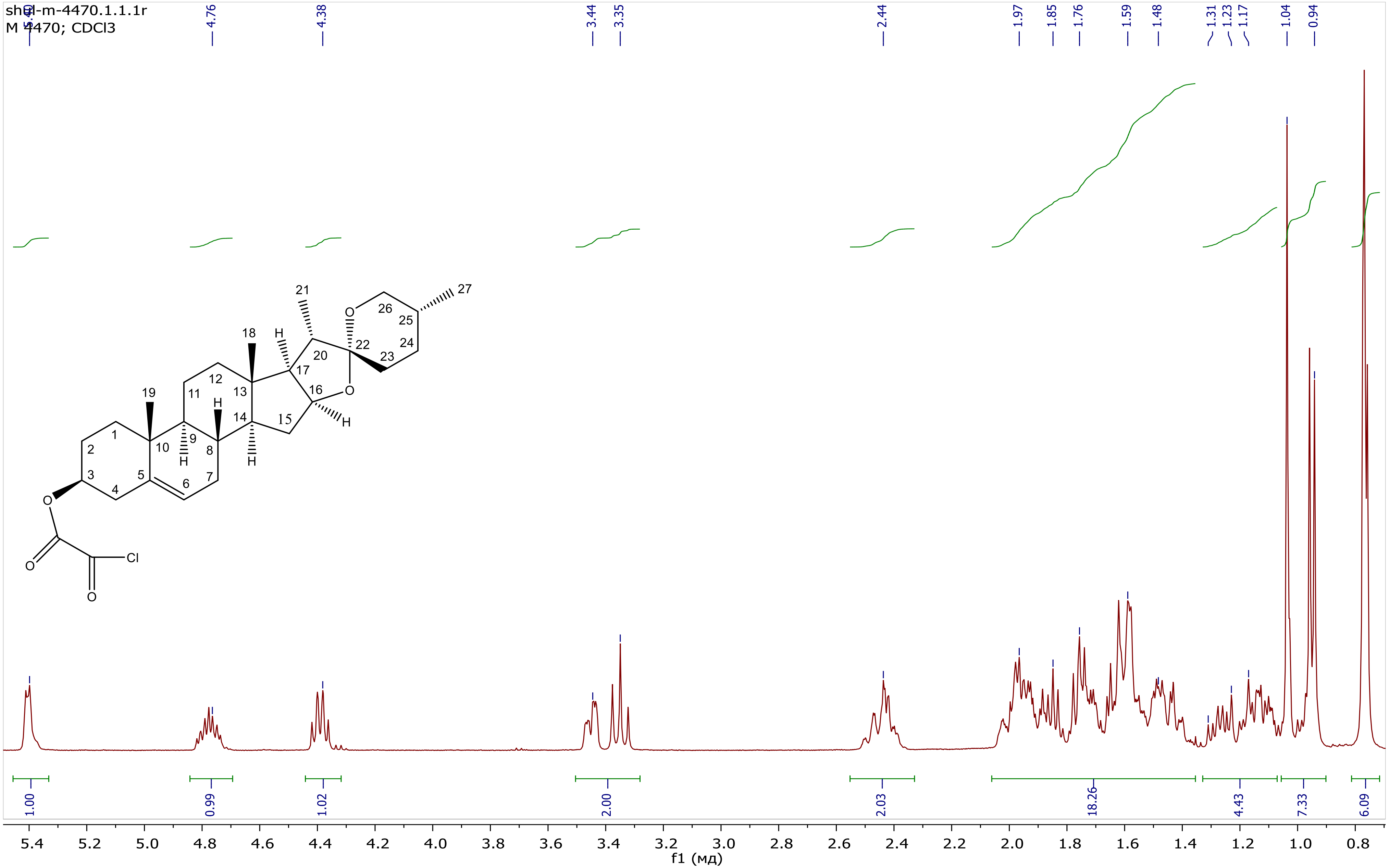

Compound 2 ((22*R*,25*R*)-spirost-5-en-3*β*-yl 2-chloro-2-oxoacetate), ^{13}C NMR, 101 MHz, $CDCl_3$

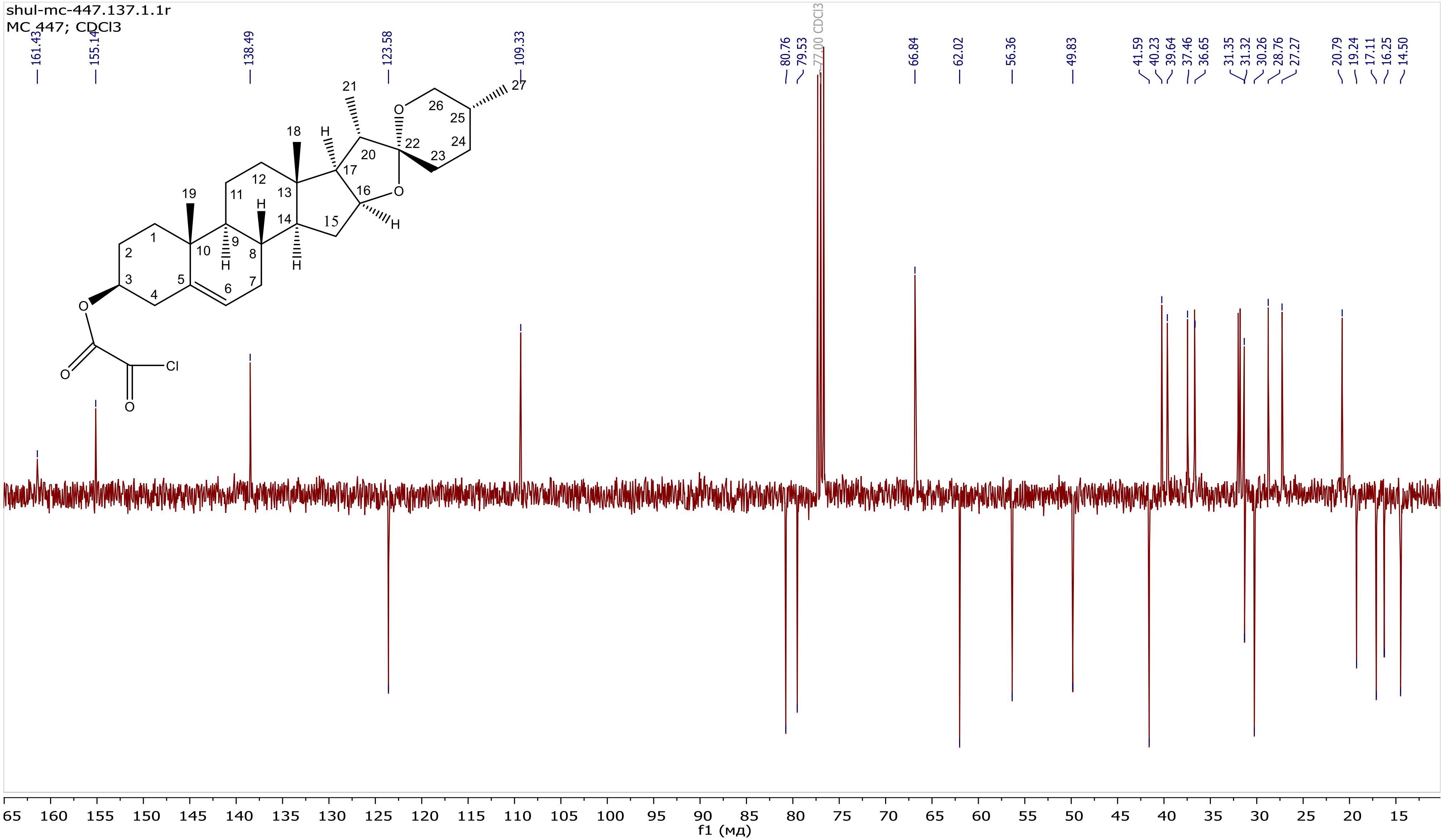

Compound 4 ((22*R*,25*R*)-spirost-5-en-3*β*-yl 2-oxo-4-phenylbut-3-ynoat), ^{1}H NMR, 600 MHz, $CDCl_3$

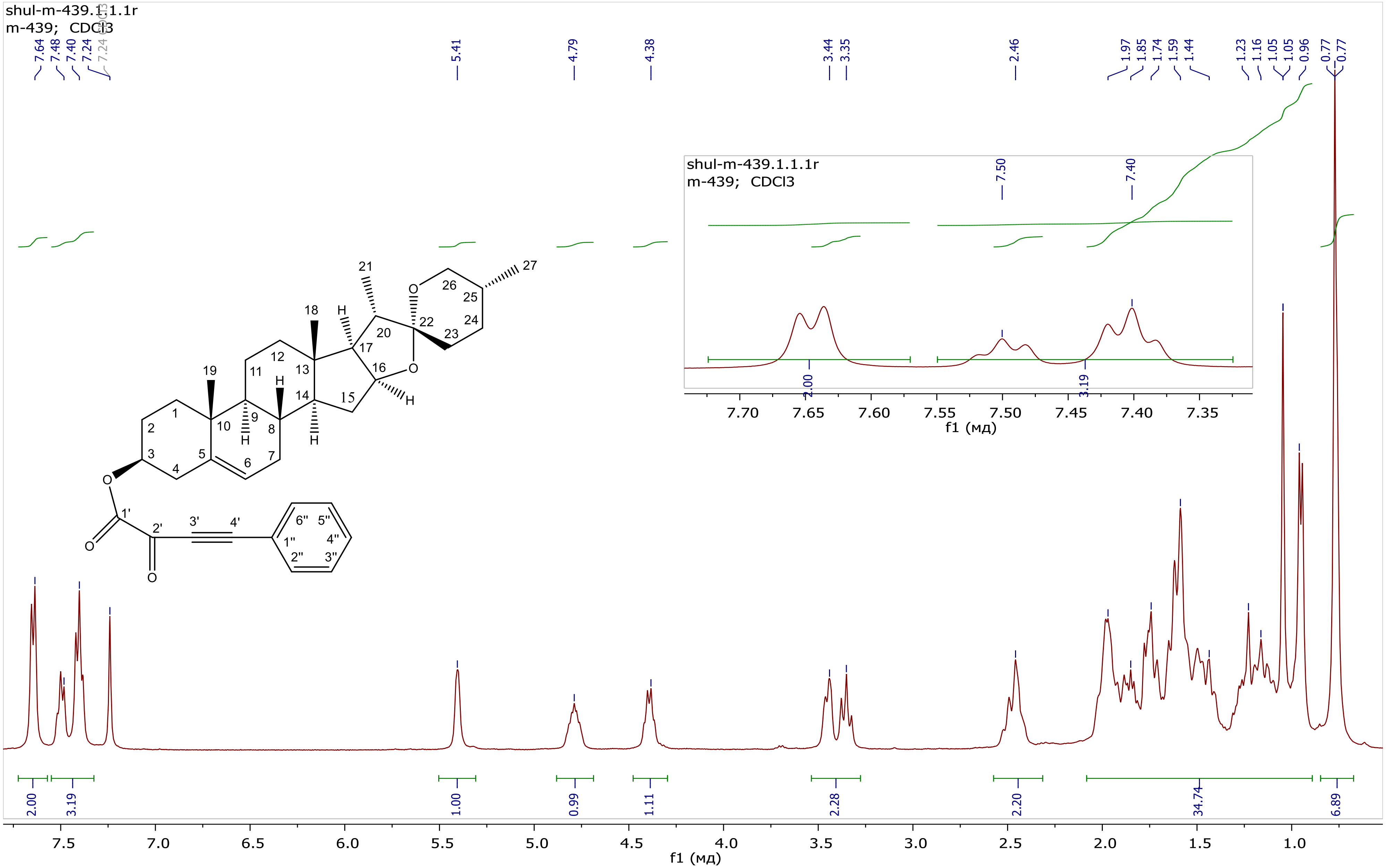

Compound 4 ((22*R*,25*R*)-spirost-5-en-3*β*-yl 2-oxo-4-phenylbut-3-ynoat), ^{13}C NMR, 151 MHz, $CDCl_3$

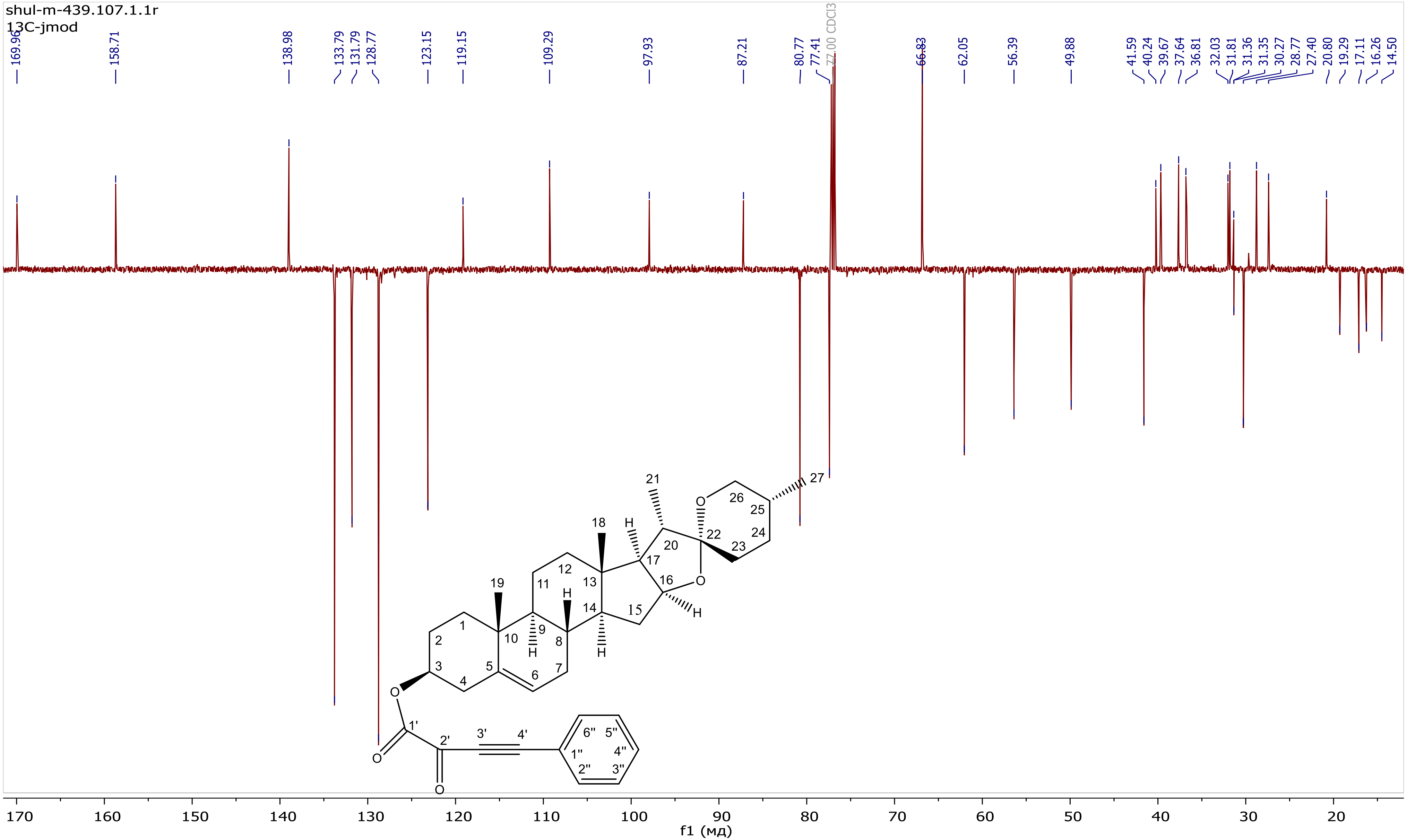

Compound 5 ((22*R*,25*R*)-spirost-5-en-3*β*-yl 4-(4-ethylphenyl)-2-oxobut-3-ynoate), ^{1}H NMR, 400 MHz, $CDCl_3$

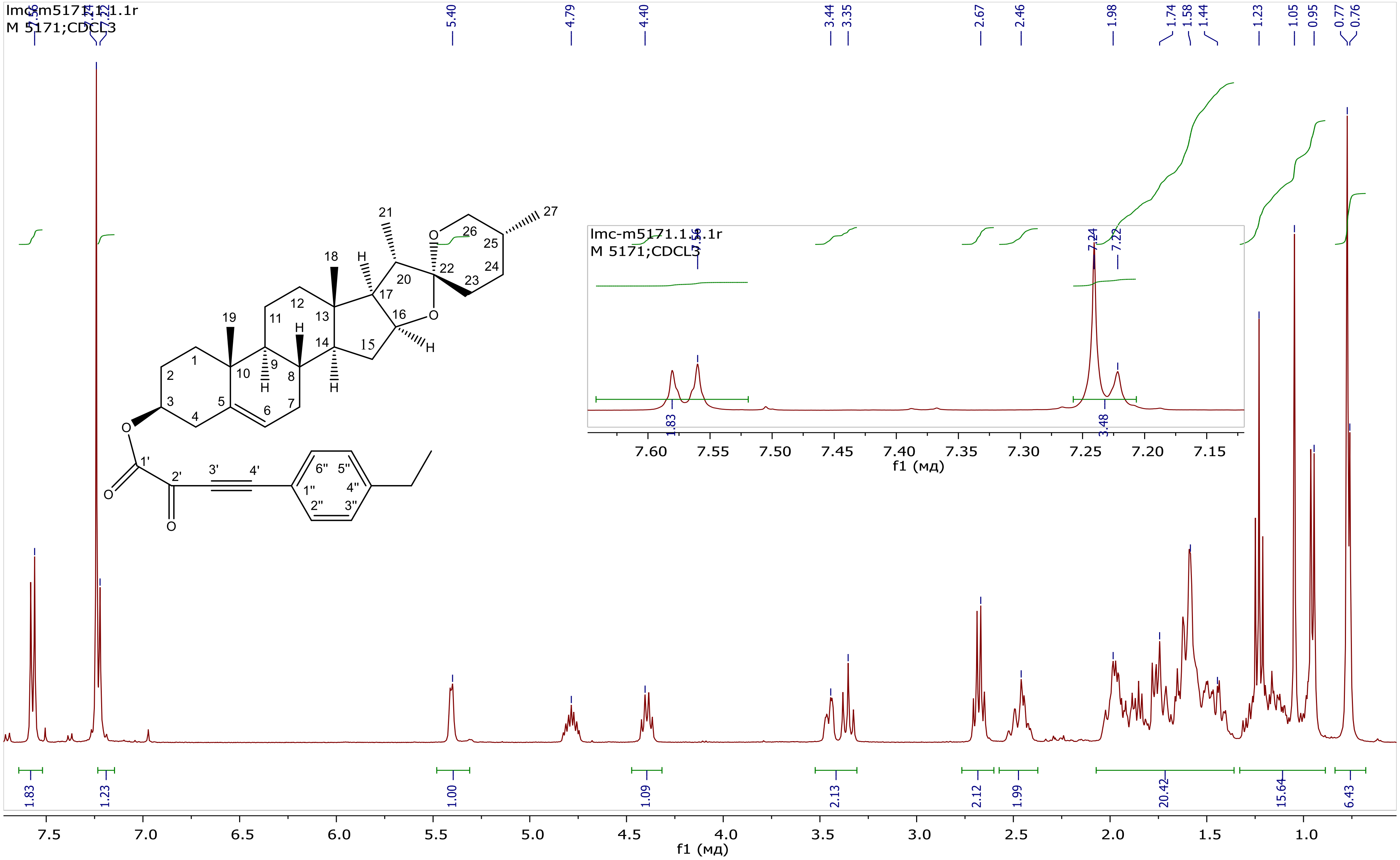

Compound 5 ((22*R*,25*R*)-spirost-5-en-3*β*-yl 4-(4-ethylphenyl)-2-oxobut-3-ynoate), ^{13}C NMR, 101 MHz, $CDCl_3$

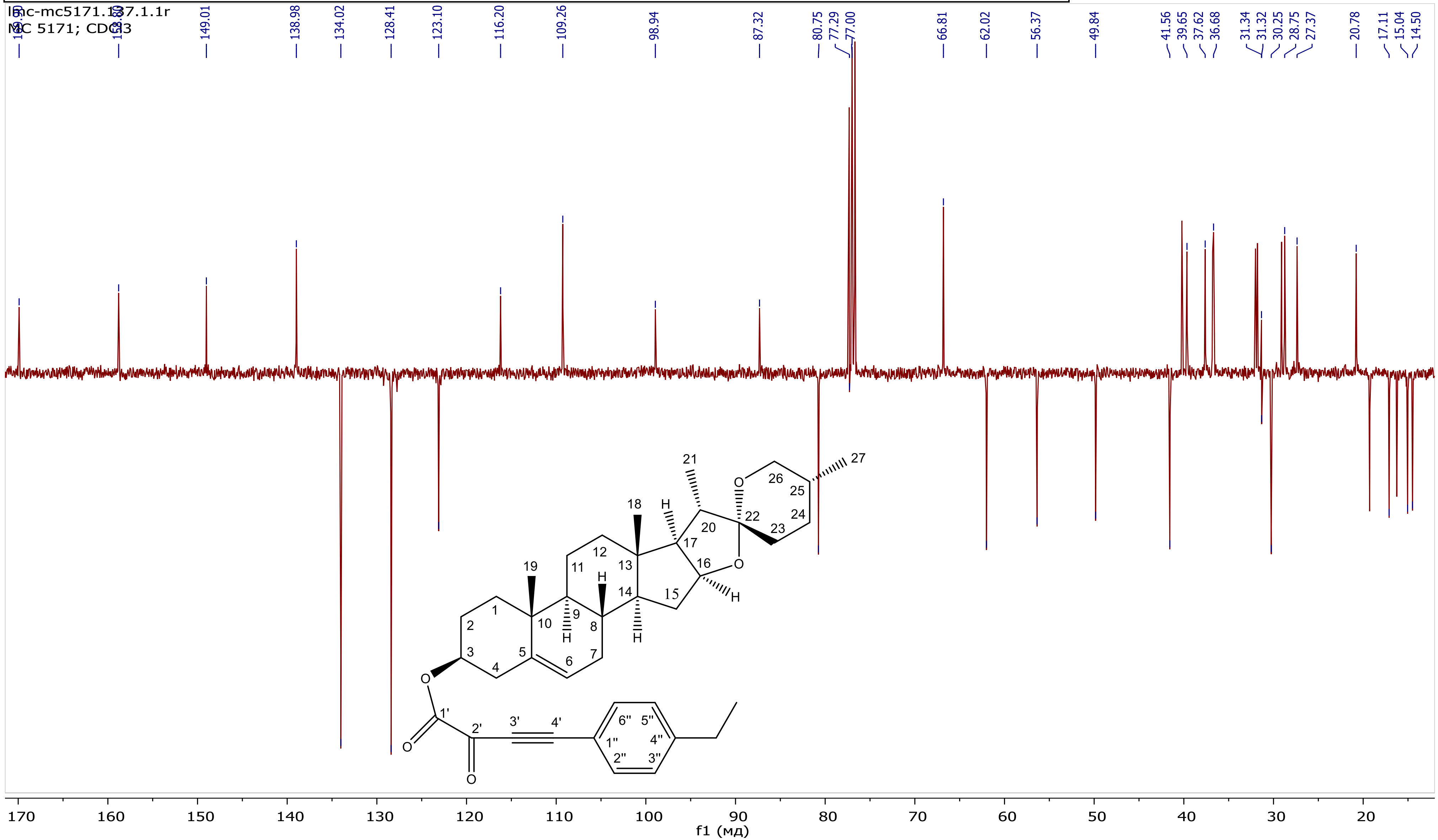

Compound 6 ((22*R*,25*R*)-spirost-5-en-3*β*-yl 4-(4-methoxyphenyl)-2-oxobut-3-ynoate), ^{1}H NMR, 400 MHz, $CDCl_3$

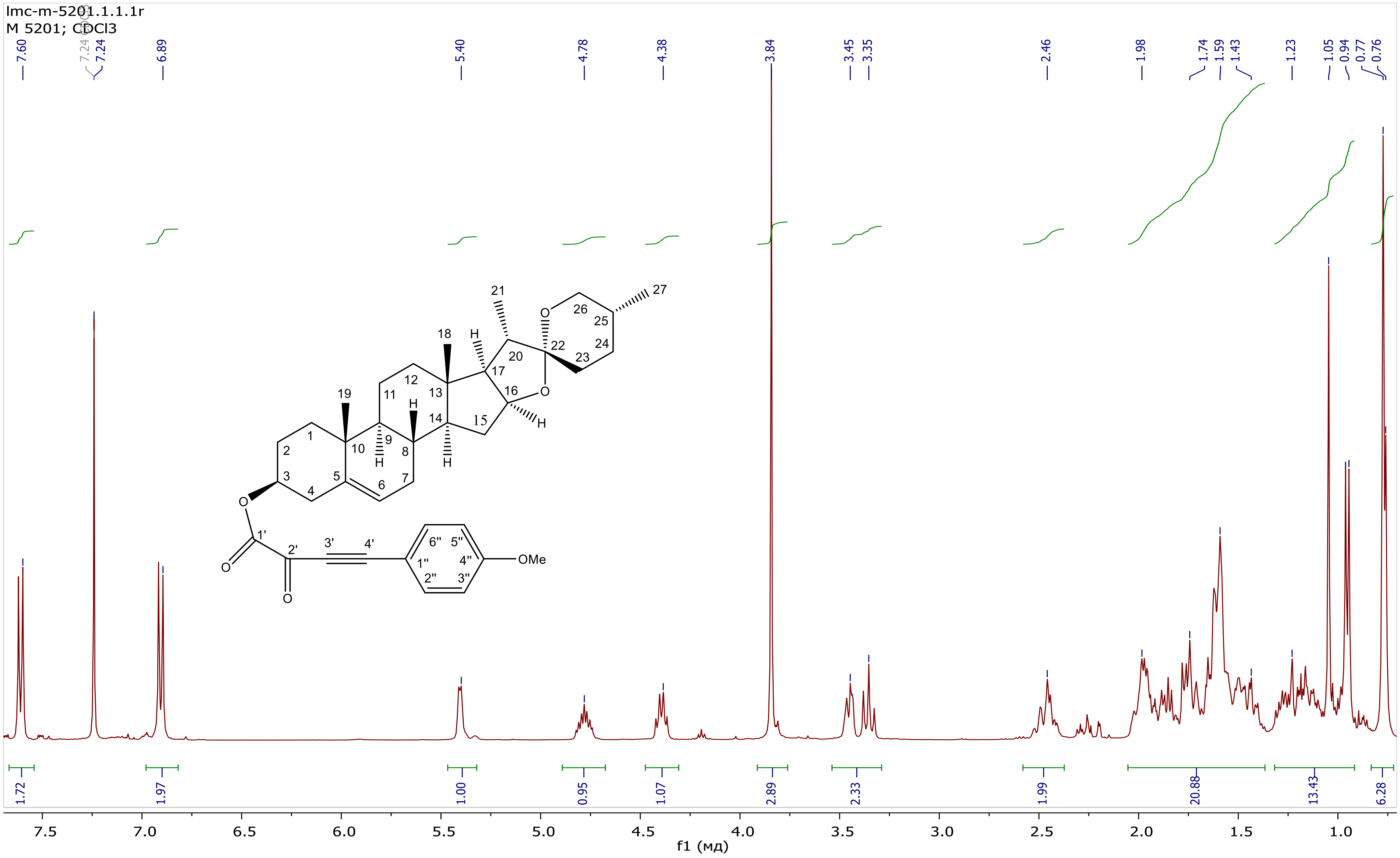

Compound 6 ((22*R*,25*R*)-spirost-5-en-3*β*-yl 4-(4-methoxyphenyl)-2-oxobut-3-ynoate), ^{13}C NMR, 101 MHz, $CDCl_3$

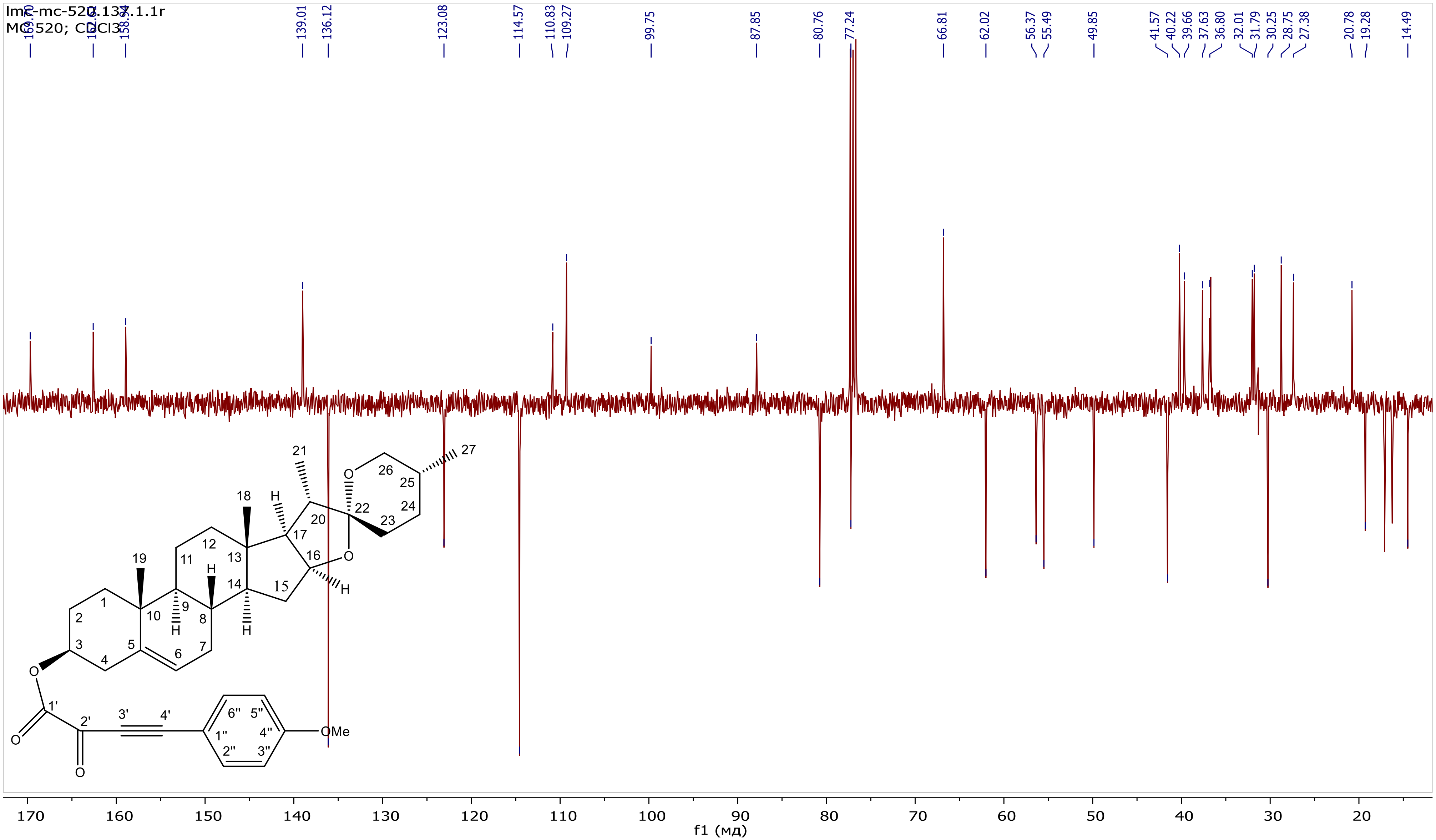

Compound 7 (22*R*,25*R*)-spirost-5-en-3*β*-yl 4-(4-fluorophenyl)-2-oxobut-3-ynoate**,** ^{1}H NMR, 400 MHz, $CDCl_3$

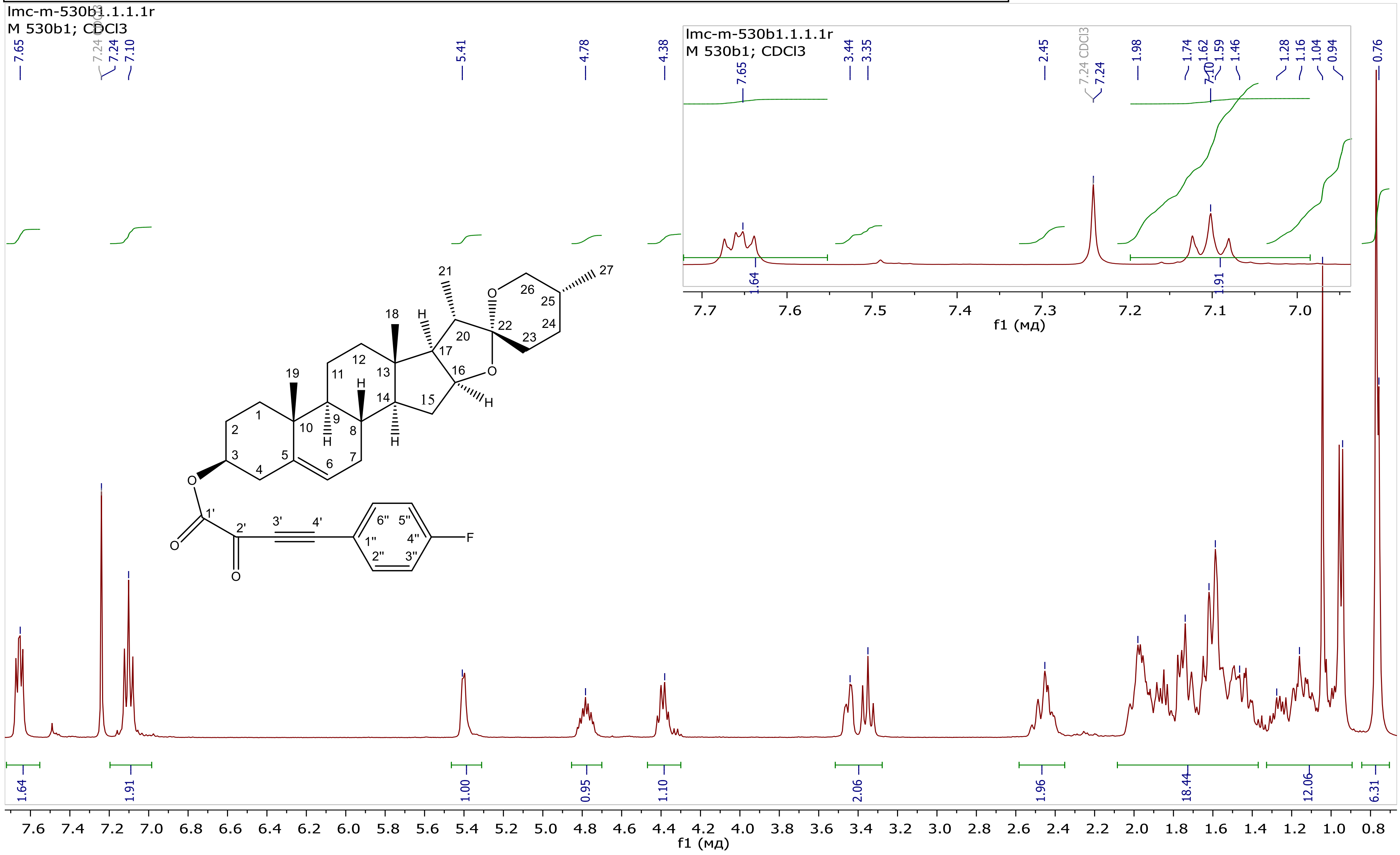

Compound 7 (22*R*,25*R*)-spirost-5-en-3*β*-yl 4-(4-fluorophenyl)-2-oxobut-3-ynoate**,** ^{13}C NMR, 126 MHz, $CDCl_3$

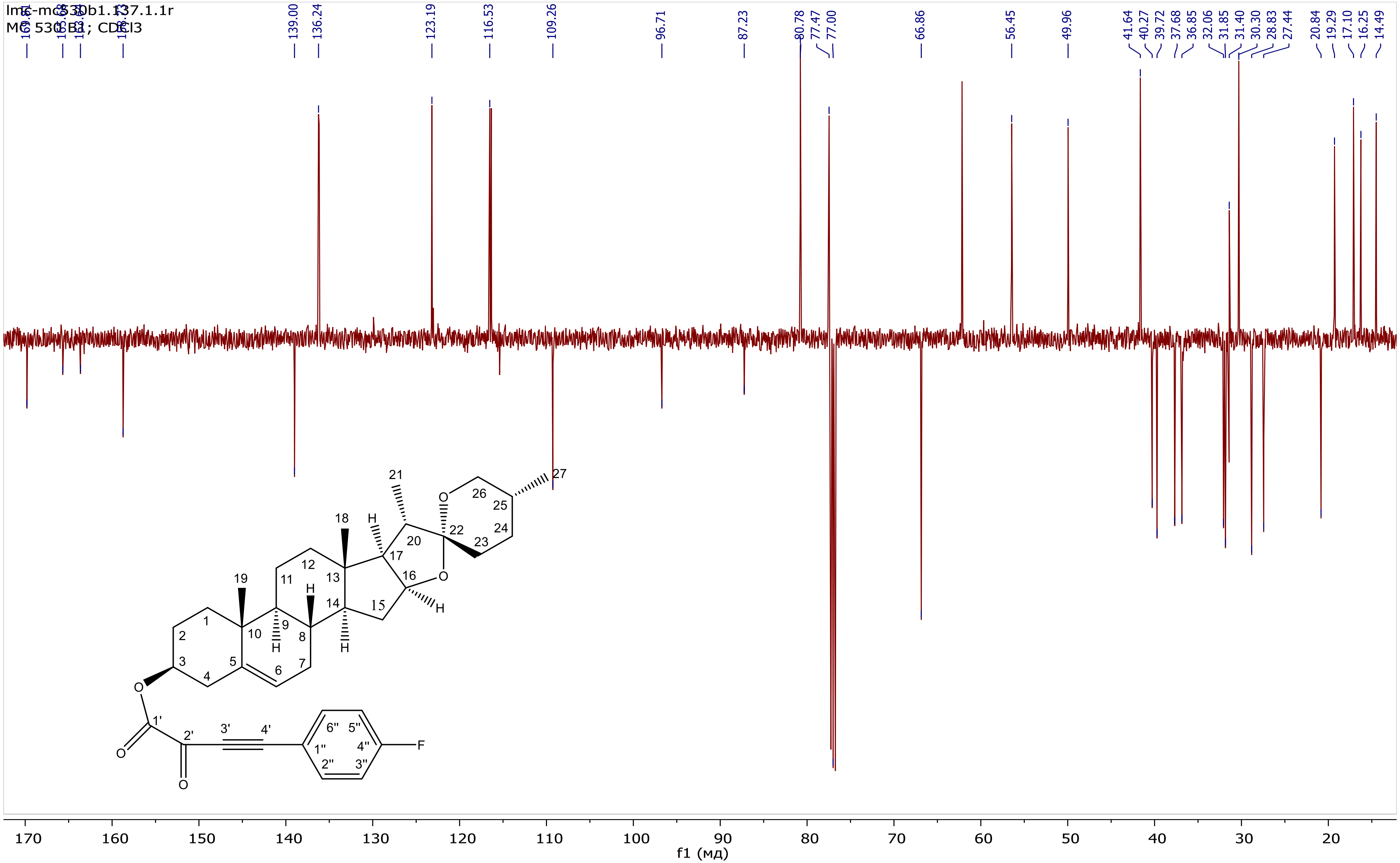

Compound 8 ((22*R*,25*R*)-spirost-5-en-3*β*-yl 2-oxo-4-(*m*-tolyl)but-3-ynoate), ^{1}H NMR, 300 MHz, $CDCl_3$

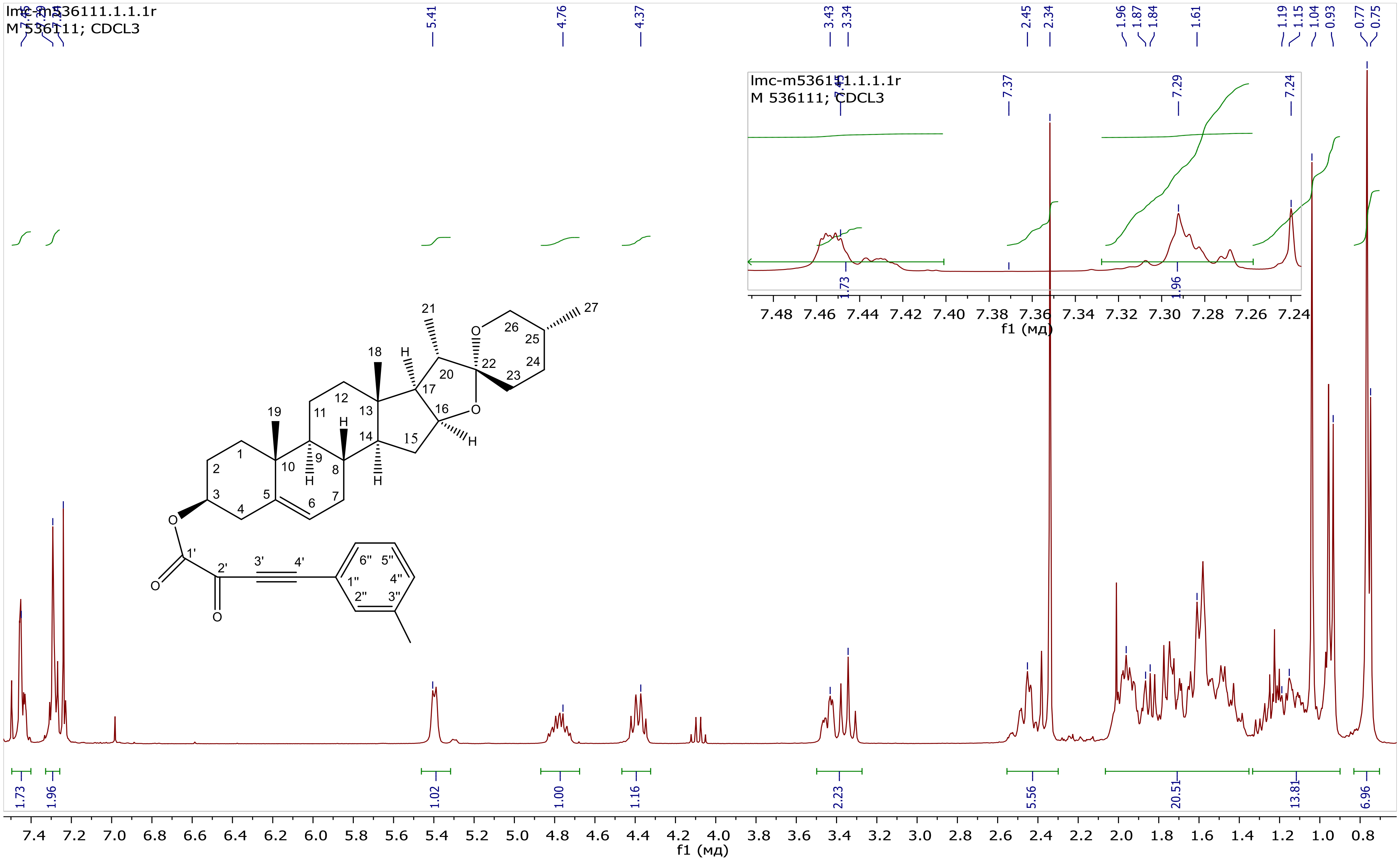

Compound 8 ((22*R*,25*R*)-spirost-5-en-3*β*-yl 2-oxo-4-(*m*-tolyl)but-3-ynoate), ^{13}C NMR, 126 MHz, $CDCl_3$

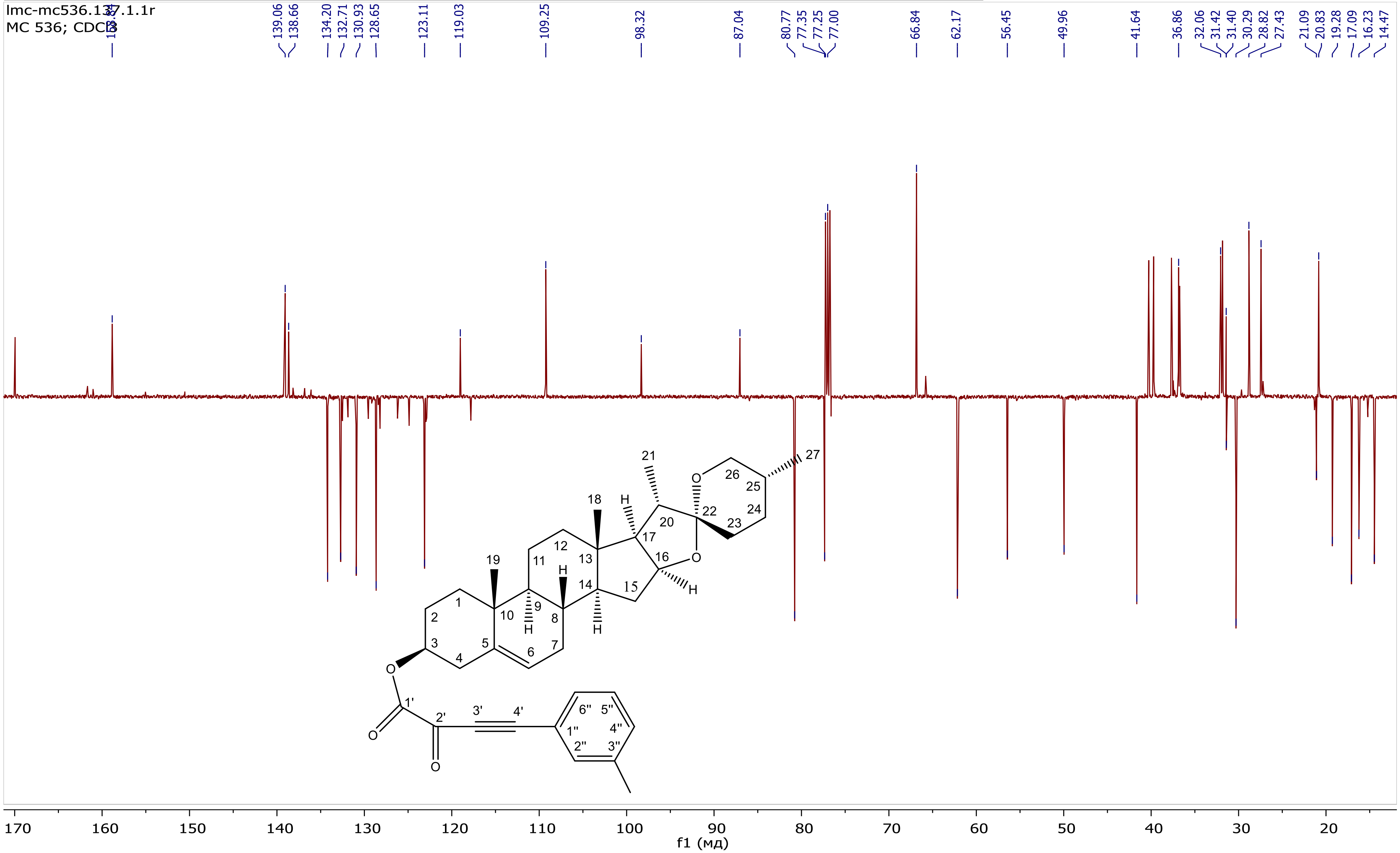

Compound 10 ((22*R*,25*R*)-spirost-5-en-3*β*-yl 1,5-diphenyl-1*H*-pyrazole-3-carboxylate), ^{1}H NMR, 600 MHz, $CDCl_3$

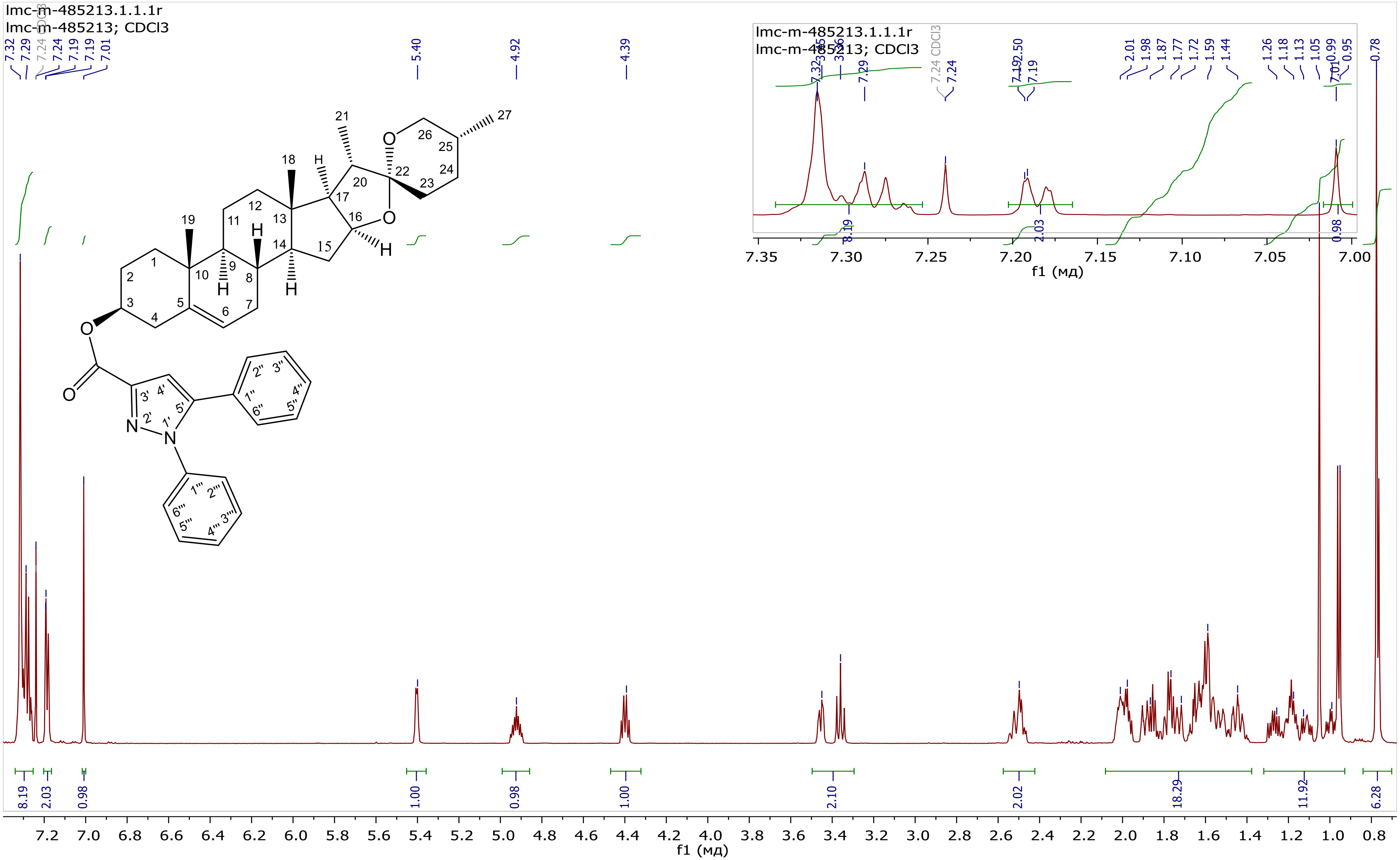

Compound 10 ((22*R*,25*R*)-spirost-5-en-3*β*-yl 1,5-diphenyl-1*H*-pyrazole-3-carboxylate), ^{13}C NMR, 151 MHz, $CDCl_3$

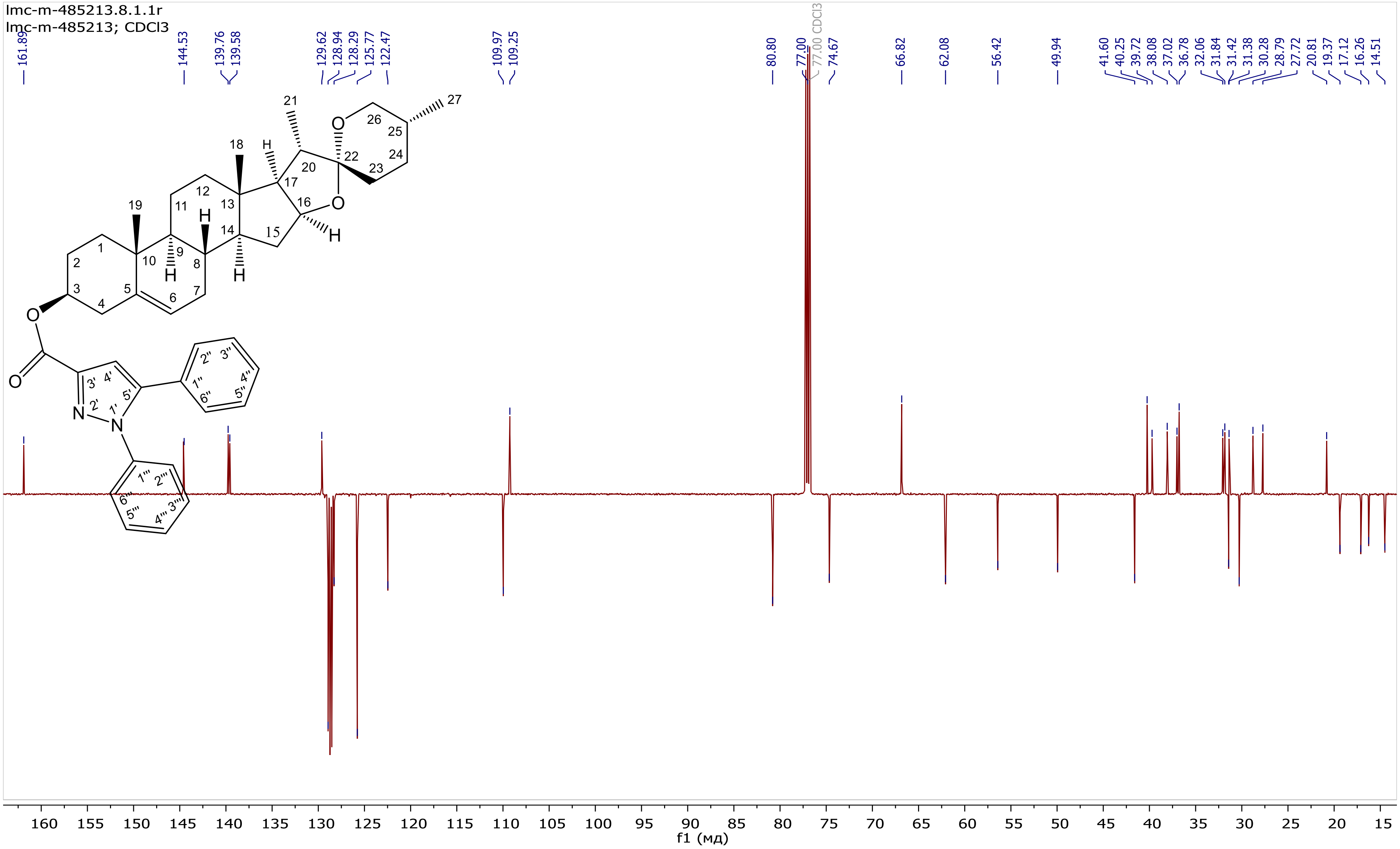

Compound 11 ((22*R*,25*R*)-spirost-5-en-3*β*-yl 5-(4-ethylphenyl)-1-phenyl-1*H*-pyrazole-3-carboxylate), ^{1}H NMR, 400 MHz, $CDCl_3$

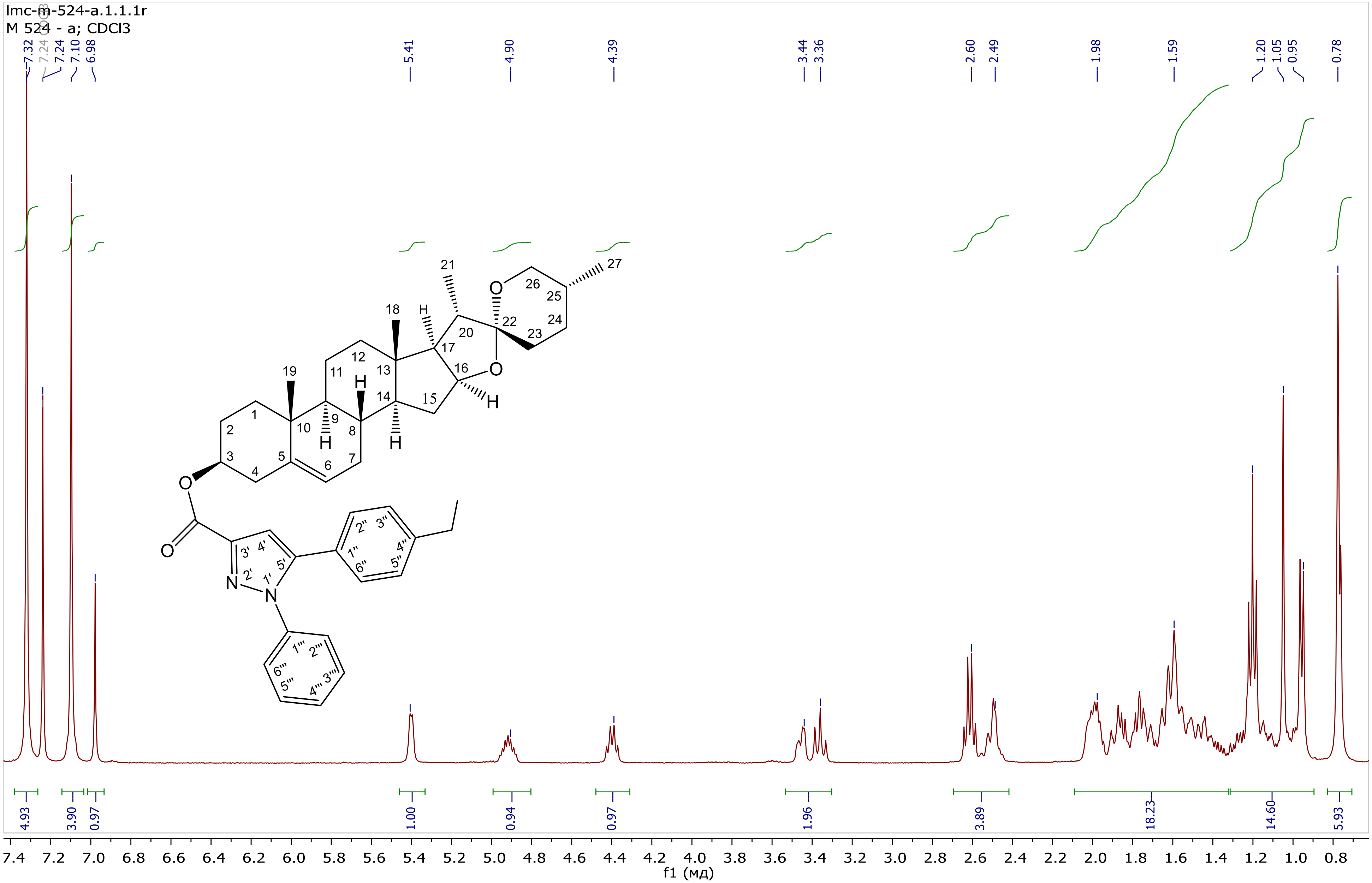

Compound 11 ((22*R*,25*R*)-spirost-5-en-3*β*-yl 5-(4-ethylphenyl)-1-phenyl-1*H*-pyrazole-3-carboxylate), ^{13}C NMR, 126 MHz, $CDCl_3$

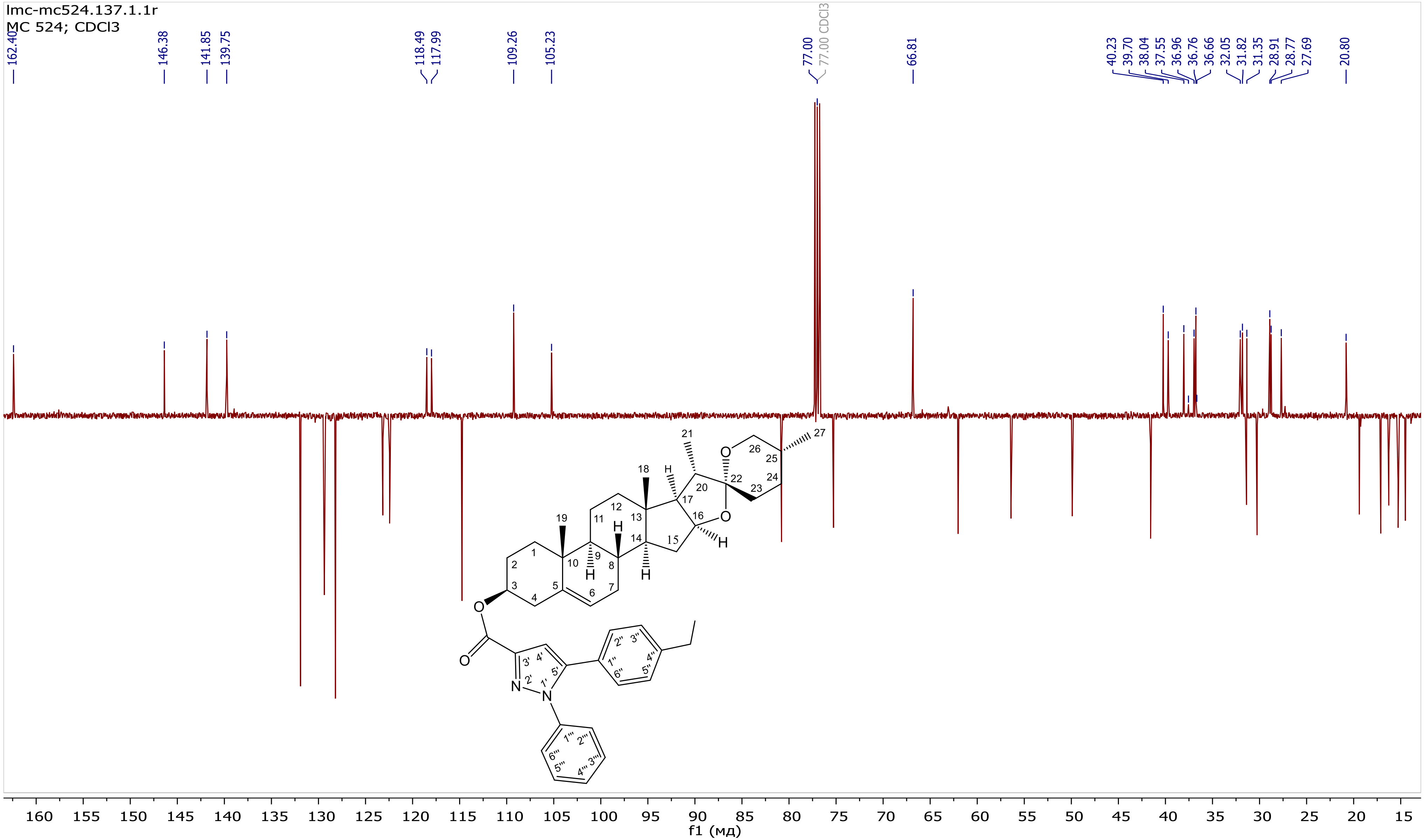

Compound 12 ((22*R*,25*R*)-spirost-5-en-3*β*-yl 5-(4-methoxyphenyl)-1-phenyl-1*H*-pyrazole-3-carboxylate), ^{1}H NMR, 400 MHz, $CDCl_3$

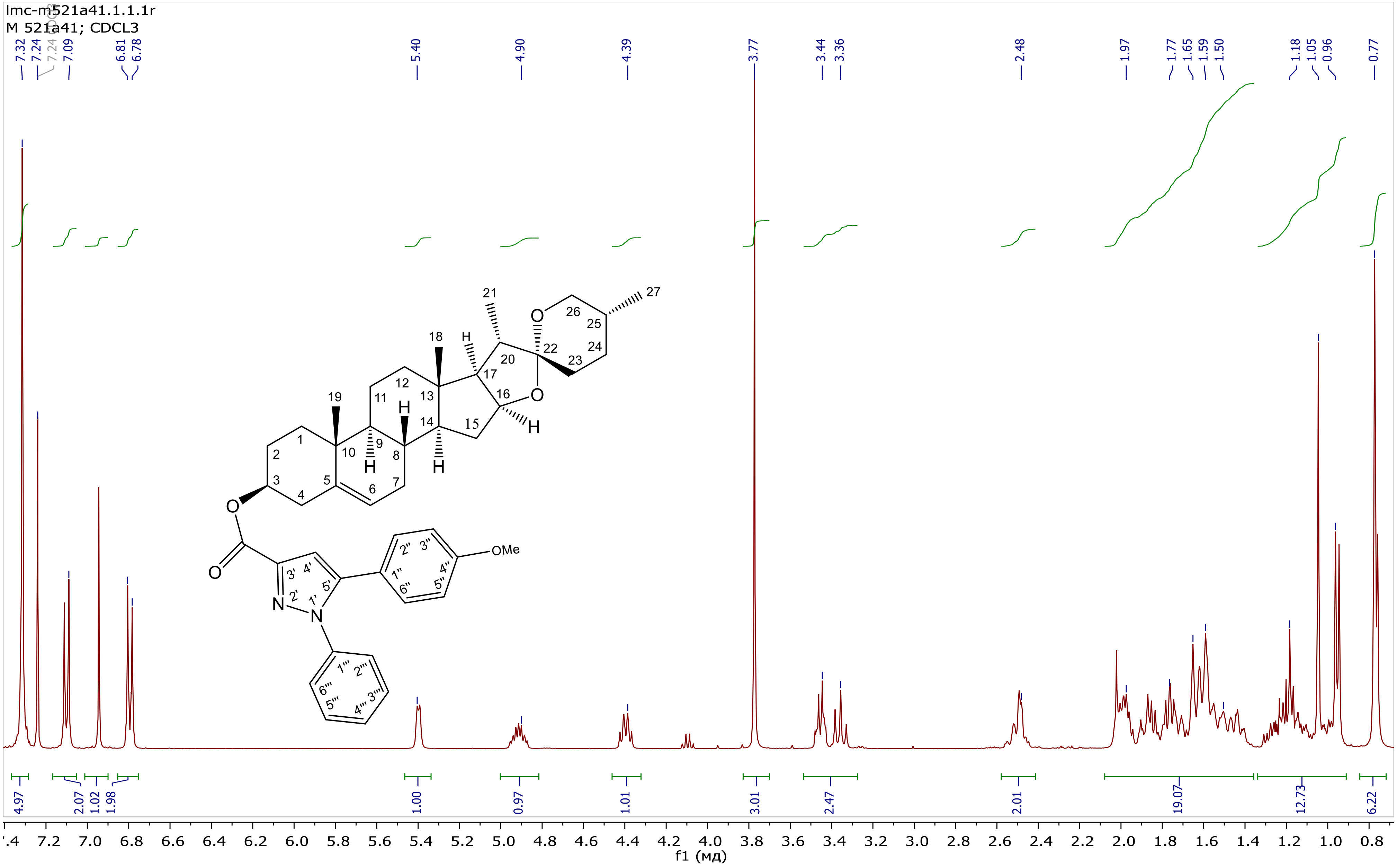

Compound 12 ((22*R*,25*R*)-spirost-5-en-3*β*-yl 5-(4-methoxyphenyl)-1-phenyl-1*H*-pyrazole-3-carboxylate), ^{13}C NMR, 126 MHz, $CDCl_3$

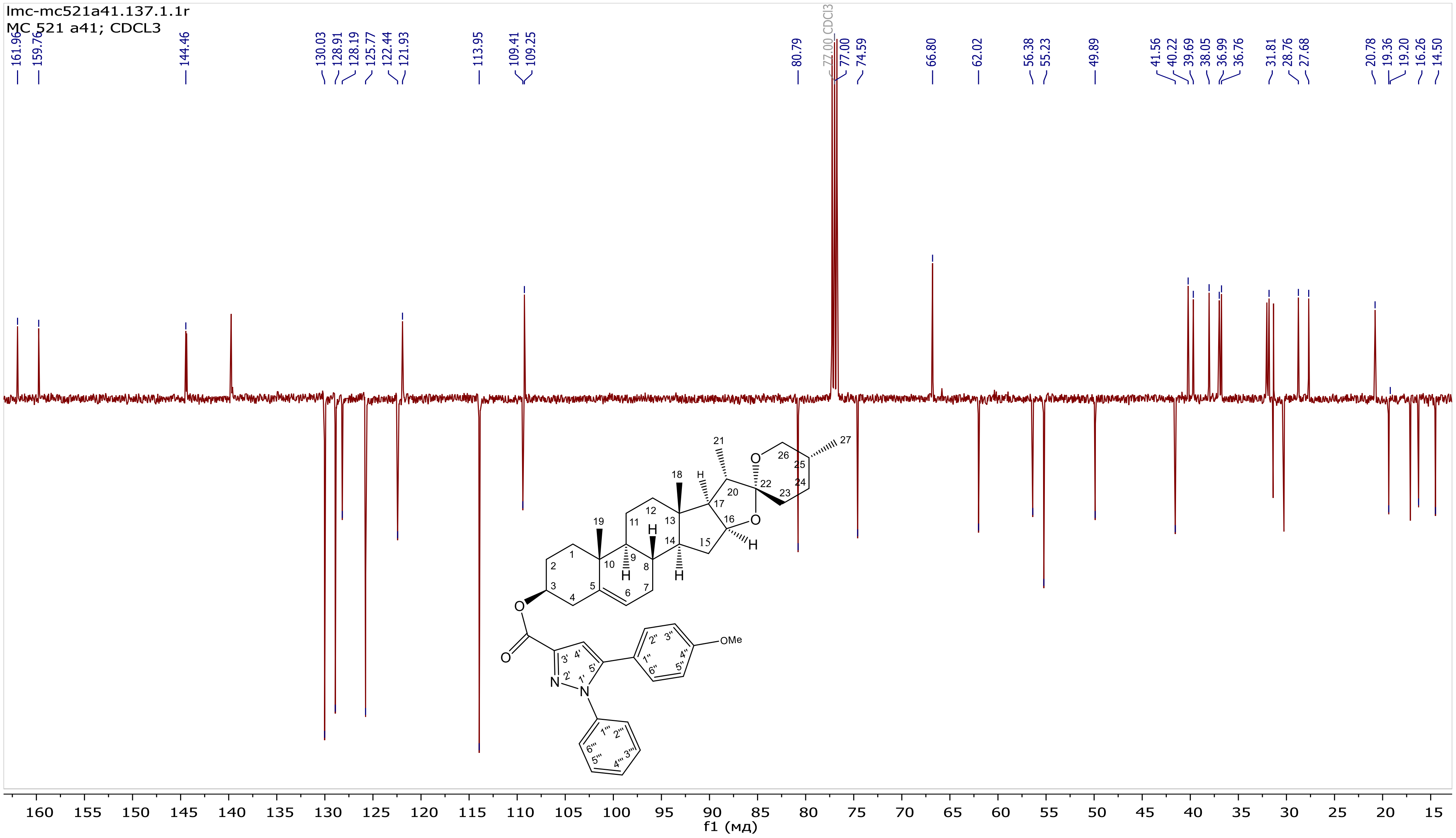

Compound 13 ((22*R*,25*R*)-spirost-5-en-3*β*-yl 5-(4-fluorophenyl)-1-phenyl-1*H*-pyrazole-3-carboxylate), ^{1}H NMR, 400 MHz, $CDCl_3$

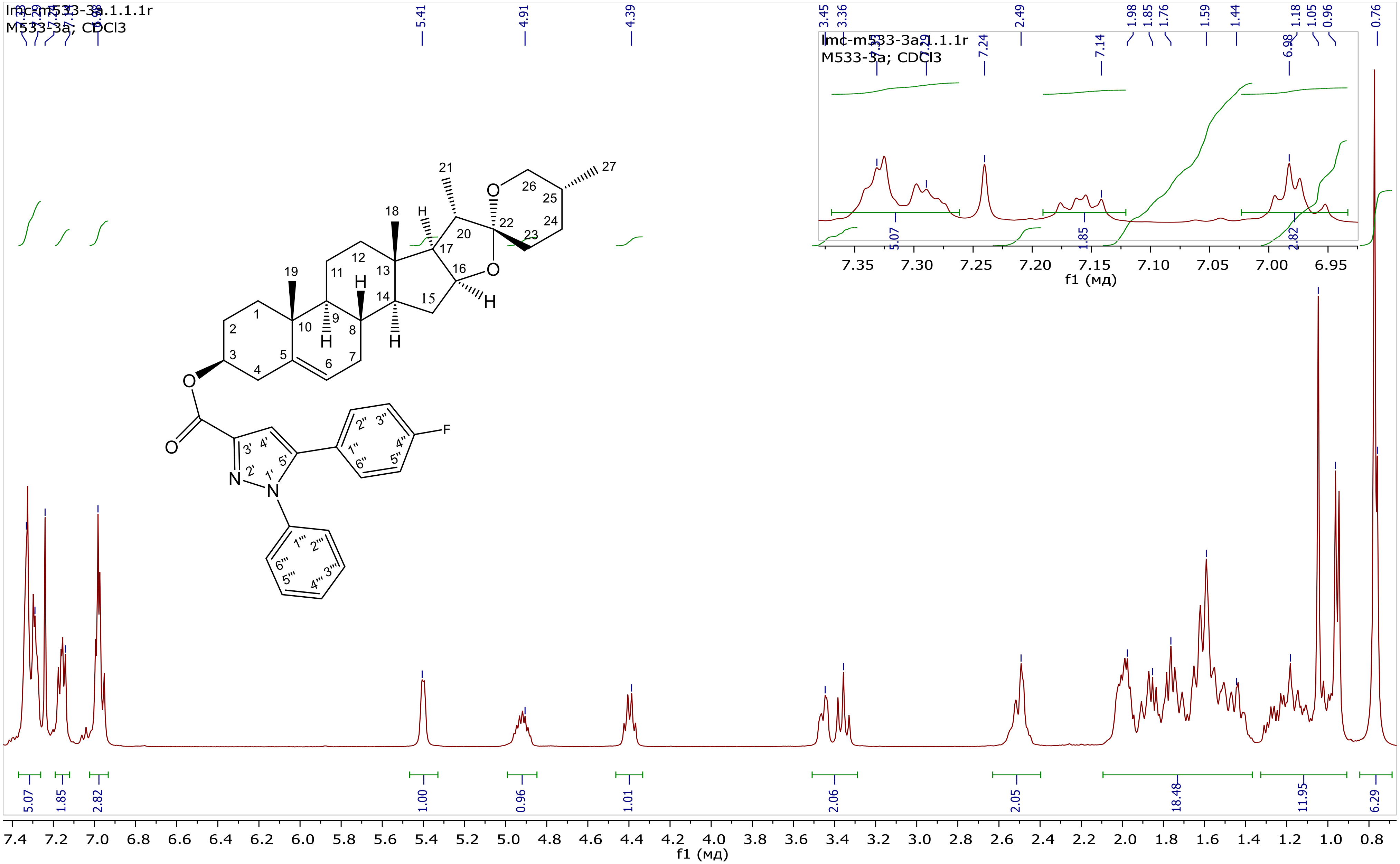

Compound 13 ((22*R*,25*R*)-spirost-5-en-3*β*-yl 5-(4-fluorophenyl)-1-phenyl-1*H*-pyrazole-3-carboxylate), ^{13}C NMR, 101 MHz, $CDCl_3$

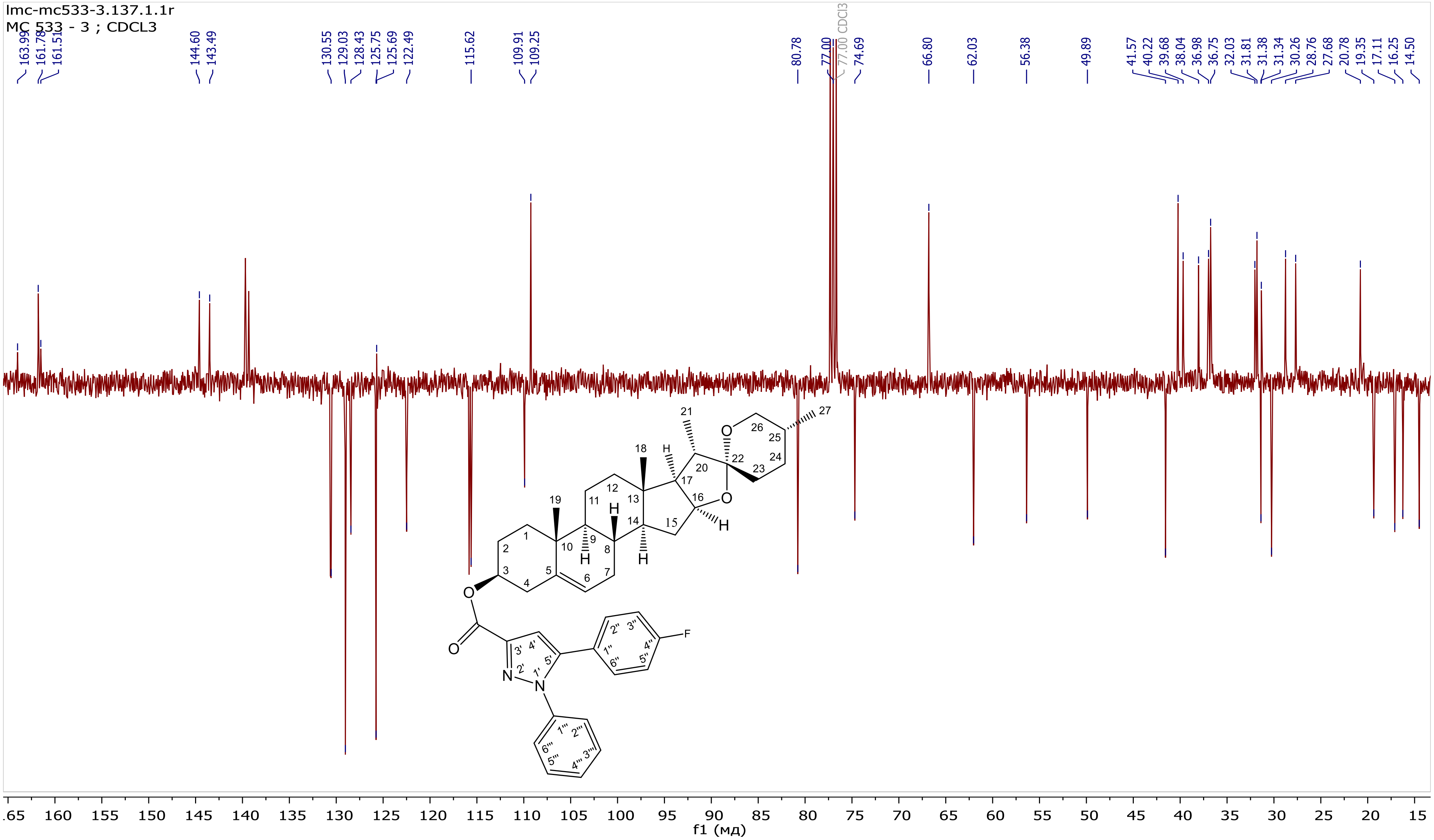

Compound 14 ((22*R*,25*R*)-spirost-5-en-3*β*-yl 1-phenyl-5-(*m*-tolyl)-1*H*-pyrazole-3-carboxylate), ^{1}H NMR, 300 MHz, $CDCl_3$

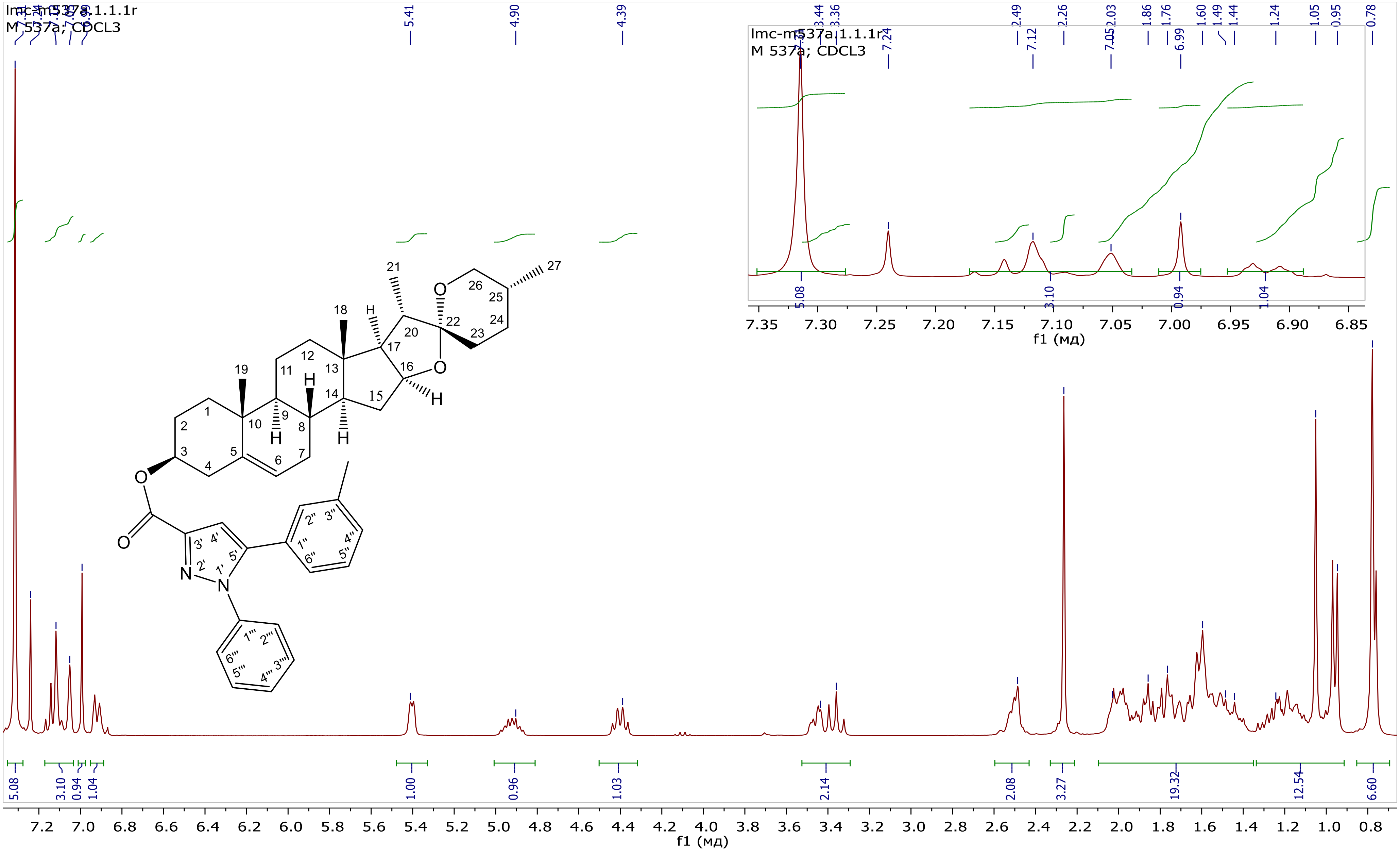

Compound 14 ((22*R*,25*R*)-spirost-5-en-3*β*-yl 1-phenyl-5-(*m*-tolyl)-1*H*-pyrazole-3-carboxylate), ^{13}C NMR, 75 MHz,

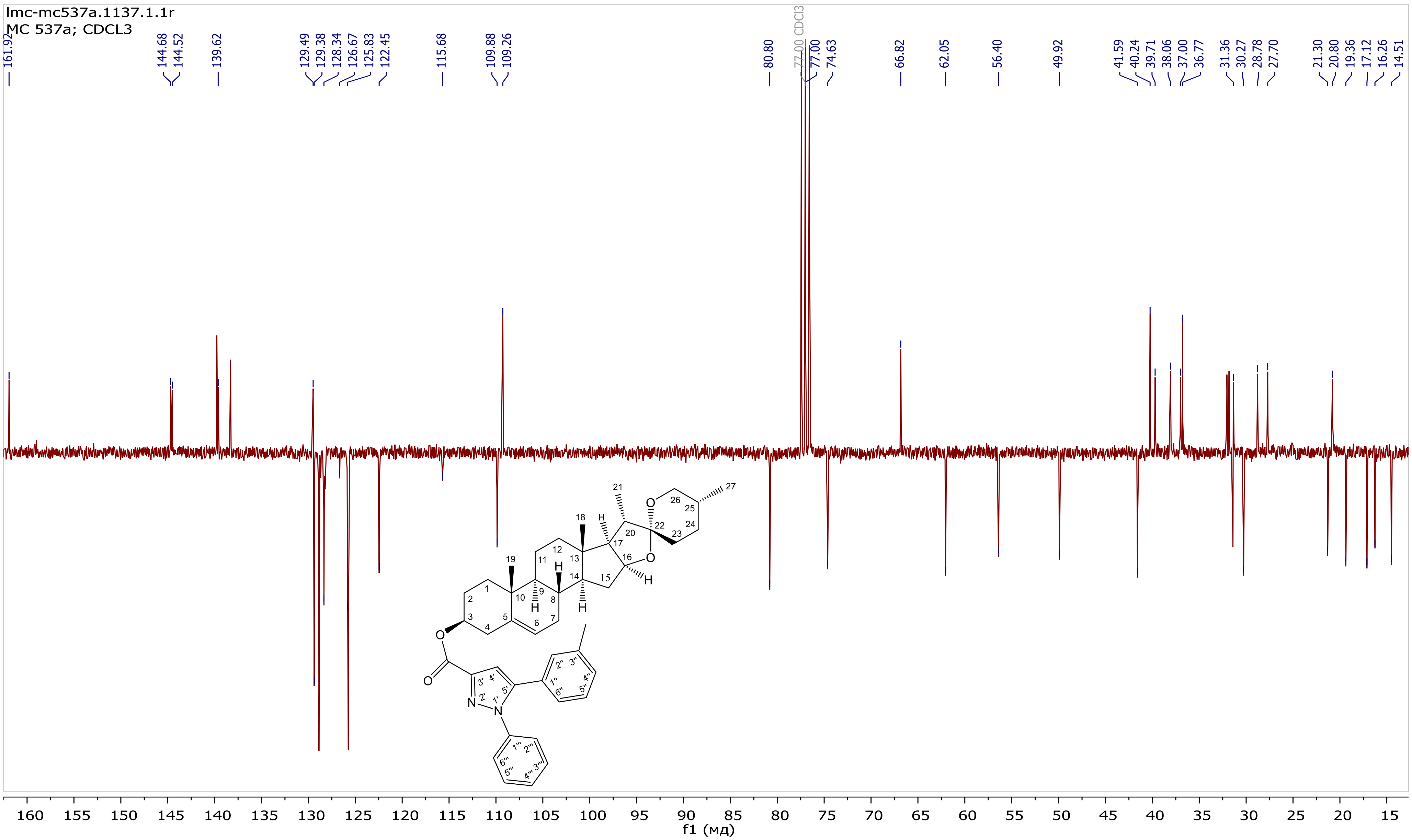

Compound 15 ((22*R*,25*R*)-spirost-5-en-3*β*-yl (*E*)-4-phenyl-2-(2-phenylhydrazineylidene)but-3-ynoate), ^{1}H NMR, 300 MHz, $CDCl_3$

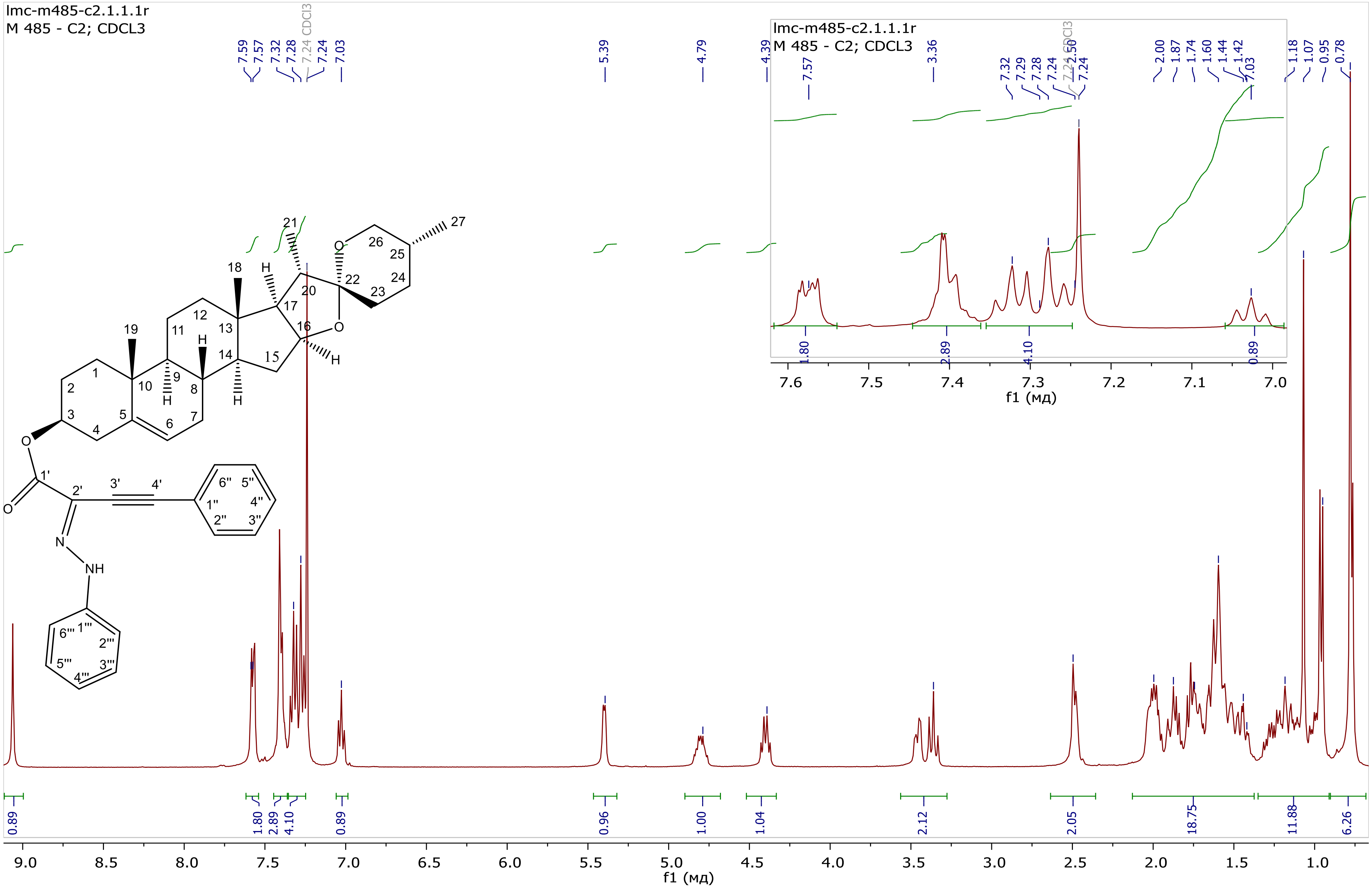

Compound 15 ((22*R*,25*R*)-spirost-5-en-3*β*-yl (*E*)-4-phenyl-2-(2-phenylhydrazineylidene)but-3-ynoate), ^{13}C NMR, 75 MHz, $CDCl_3$

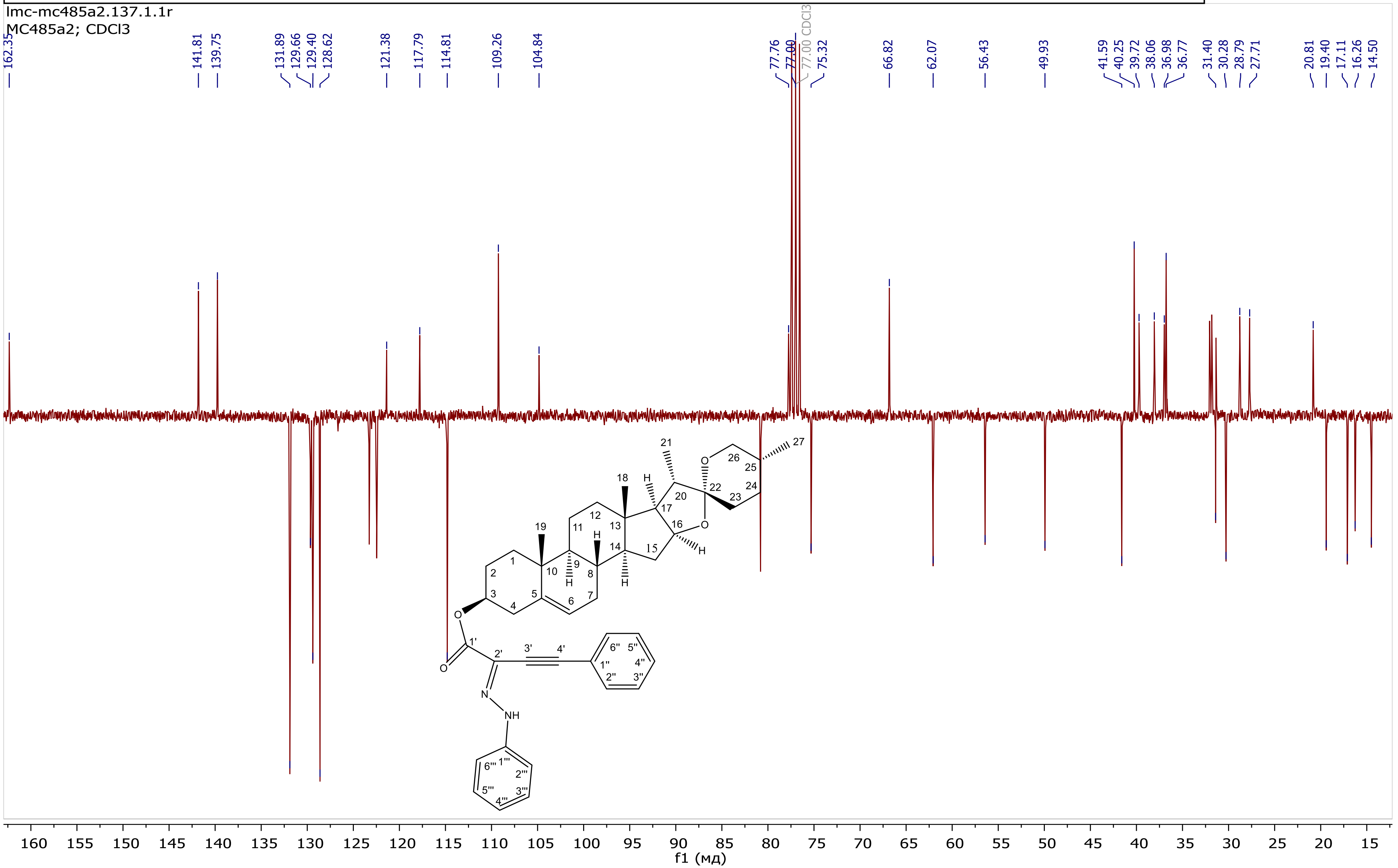

Compound 16 ((22*R*,25*R*)-spirost-5-en-3*β*-yl (*E*)-4-(4-ethylphenyl)-2-(2-phenylhydrazineylidene)but-3-ynoate), ^{1}H NMR, 600 MHz, $CDCl_3$

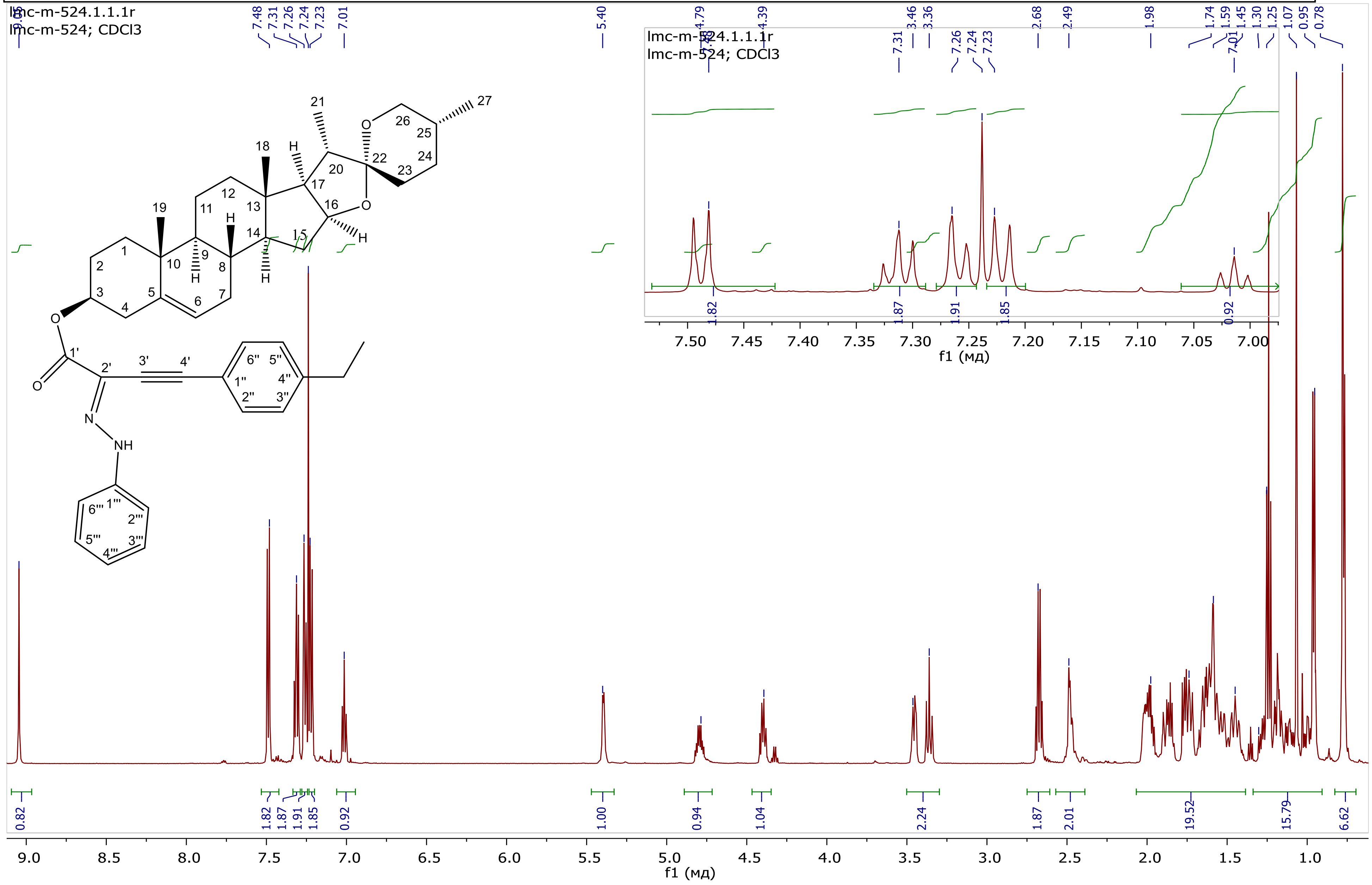

Compound 16 ((22*R*,25*R*)-spirost-5-en-3*β*-yl (*E*)-4-(4-ethylphenyl)-2-(2-phenylhydrazineylidene)but-3-ynoate), ^{13}C NMR, 151 MHz, $CDCl_3$

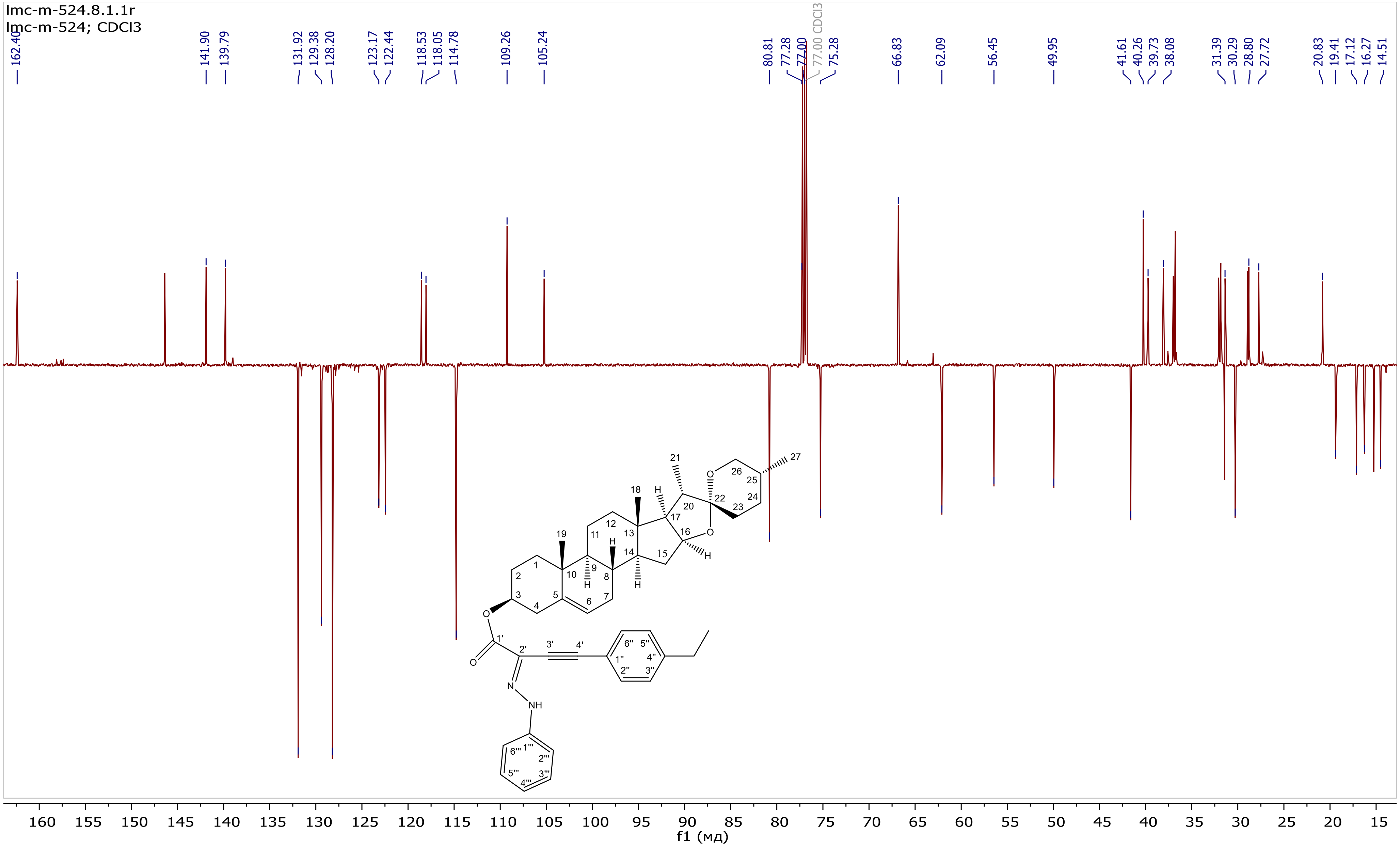

Compound 17 ((22*R*,25*R*)-spirost-5-en-3*β*-yl (*E*)-4-(4-methoxyhenyl)-2-(2-phenylhydrazineylidene)but-3-ynoate), ^{1}H NMR, 600 MHz, $CDCl_3$

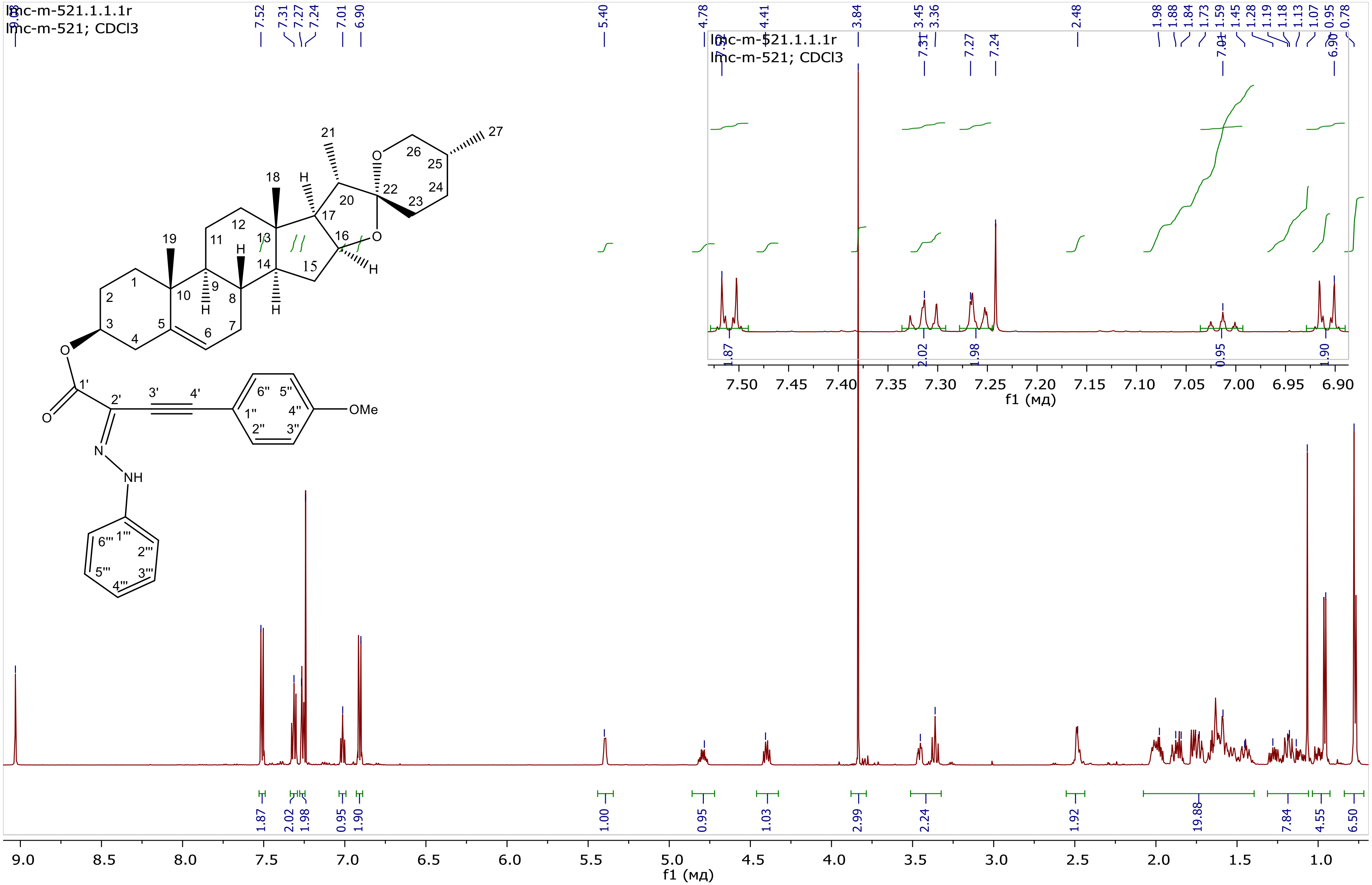

Compound 17 ((22*R*,25*R*)-spirost-5-en-3*β*-yl (*E*)-4-(4-methoxyhenyl)-2-(2-phenylhydrazineylidene)but-3-ynoate), ^{13}C NMR, 151 MHz, $CDCl_3$

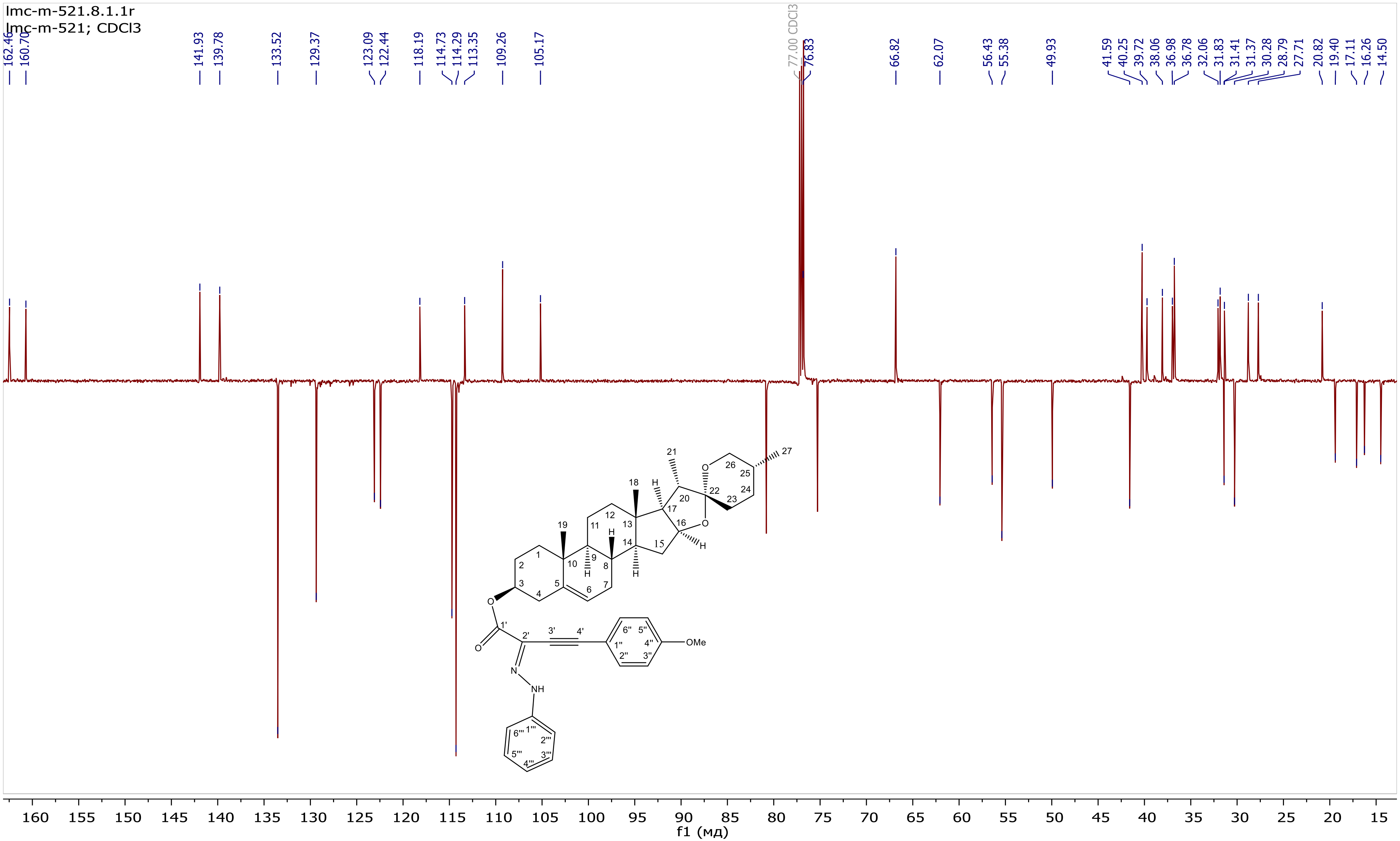

Compound 18 (22*R*,25*R*)-spirost-5-en-3*β*-yl (*E*)-4-(4-fluorophenyl)-2-(2-phenylhydrazineylidene)but-3-ynoate, ^{1}H NMR, 400 MHz, $CDCl_3$

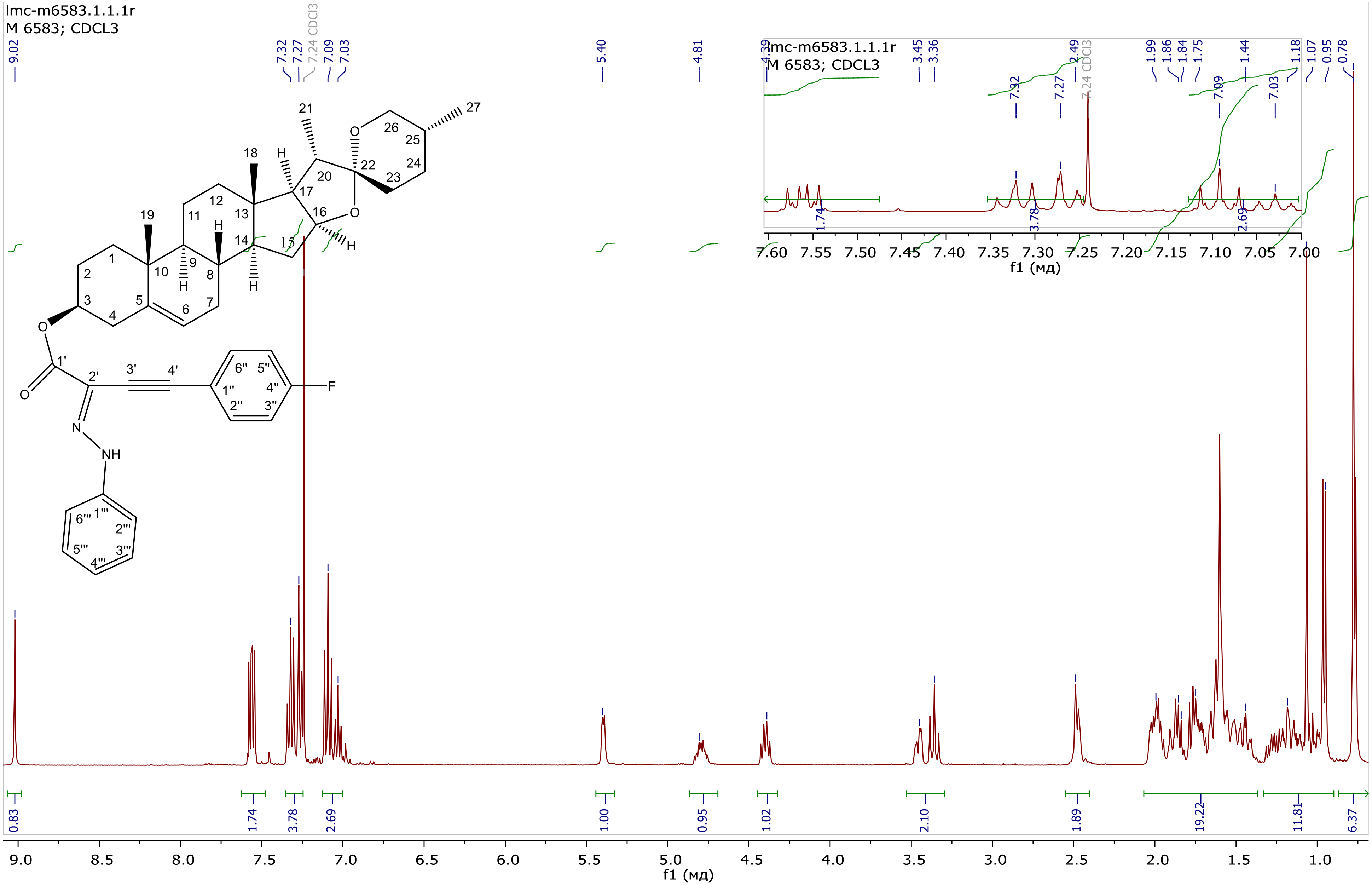

Compound 18 (22*R*,25*R*)-spirost-5-en-3*β*-yl (*E*)-4-(4-fluorophenyl)-2-(2-phenylhydrazineylidene)but-3-ynoate, ^{13}C NMR, 101 MHz, $CDCl_3$

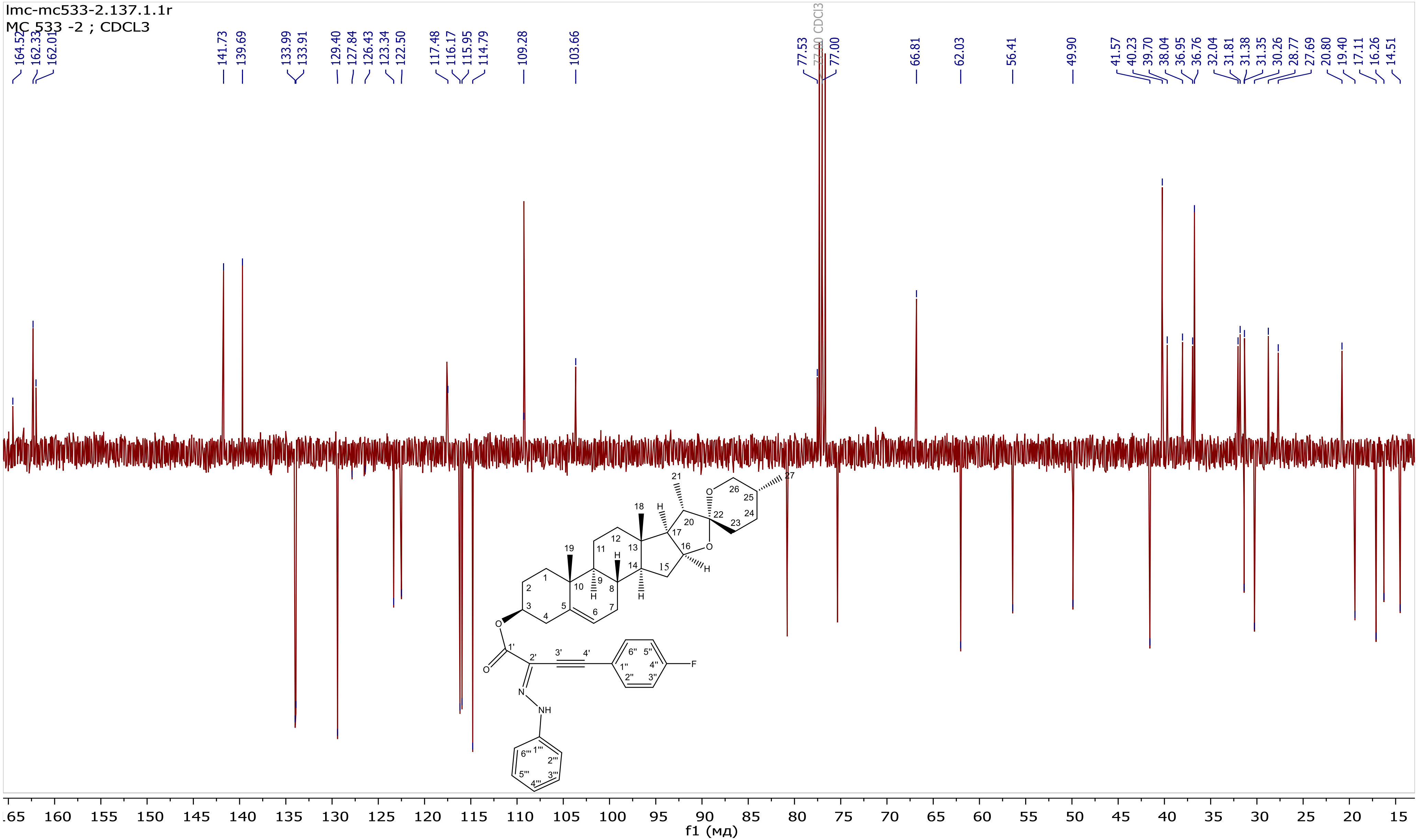

Compound 19 ((22*R*,25*R*)-spirost-5-en-3β-yl (*E*)-2-(2-phenylhydrazineylidene)-4-(*m*-tolyl)but-3-ynoate), ^{1}H NMR, 500 MHz, $CDCl_3$

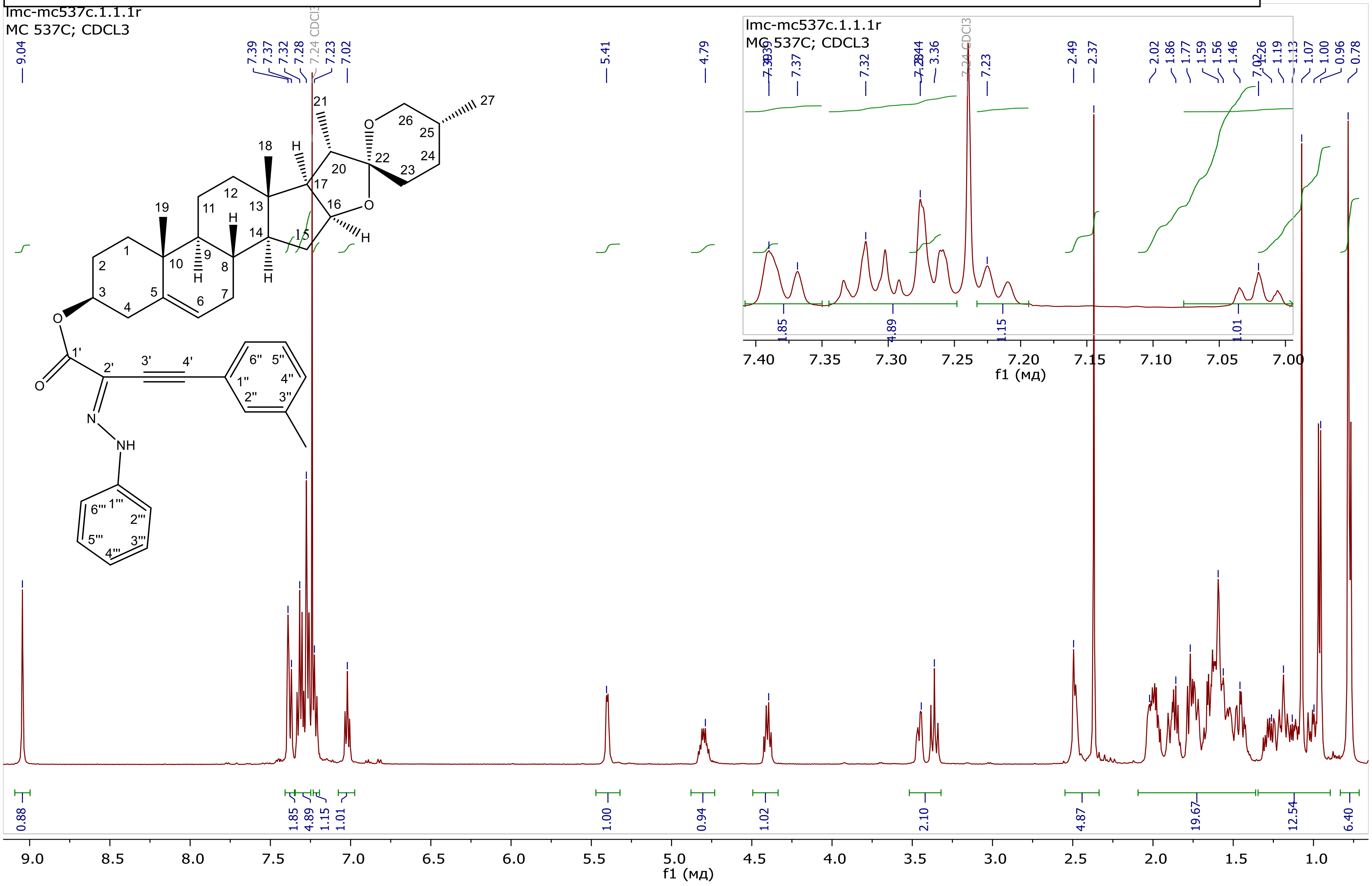

Compound 19 ((22*R*,25*R*)-spirost-5-en-3*β*-yl (*E*)-2-(2-phenylhydrazineylidene)-4-(*m*-tolyl)but-3-ynoate), ^{13}C NMR, 126 MHz, CDCl$_3$

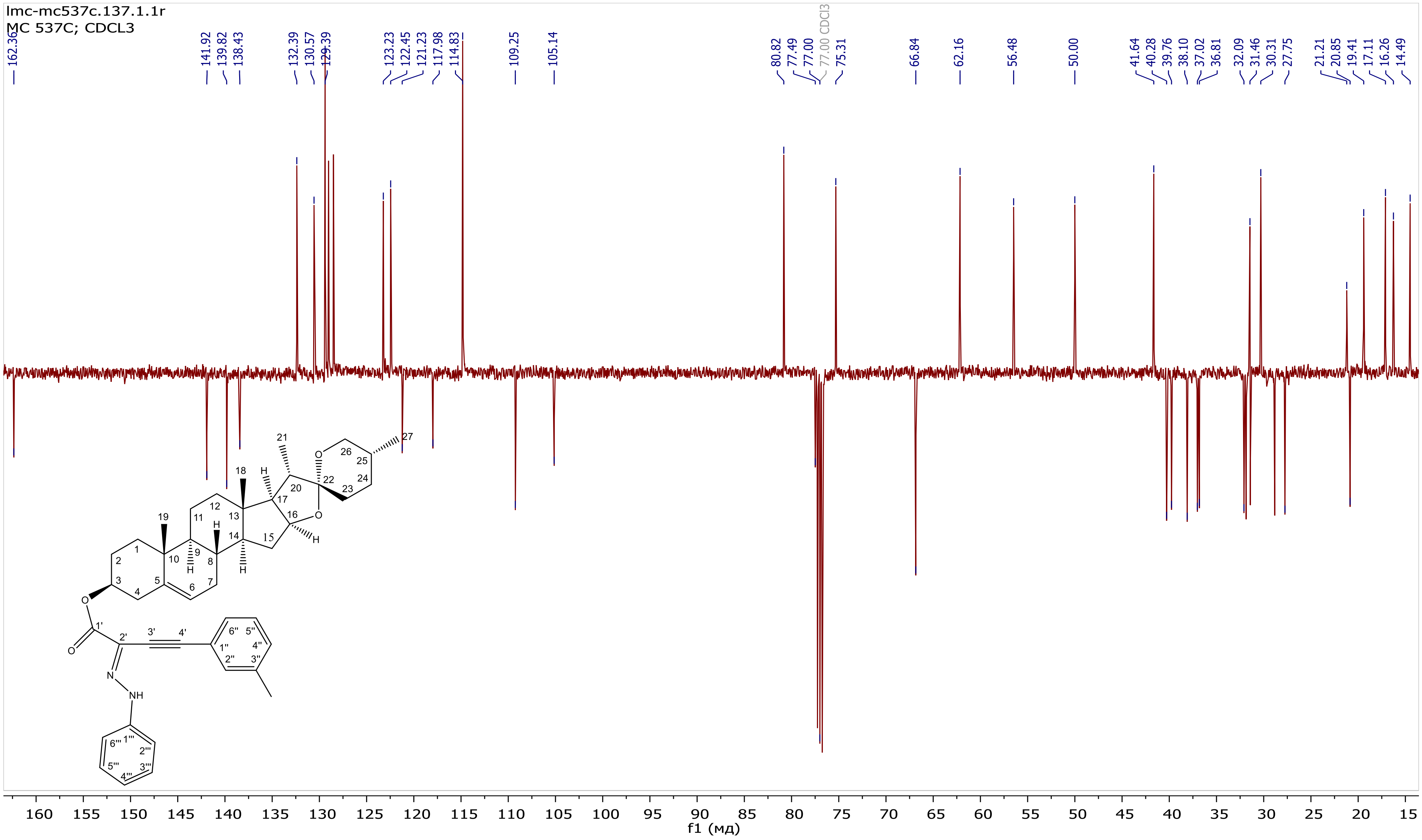

Compound 20 ((22*R*,25*R*)-spirost-5-en-3*β*-yl (*Z*)-4-phenyl-2-(2-phenylhydrazineylidene)but-3-ynoate), ^{1}H NMR, 300 MHz, $CDCl_3$

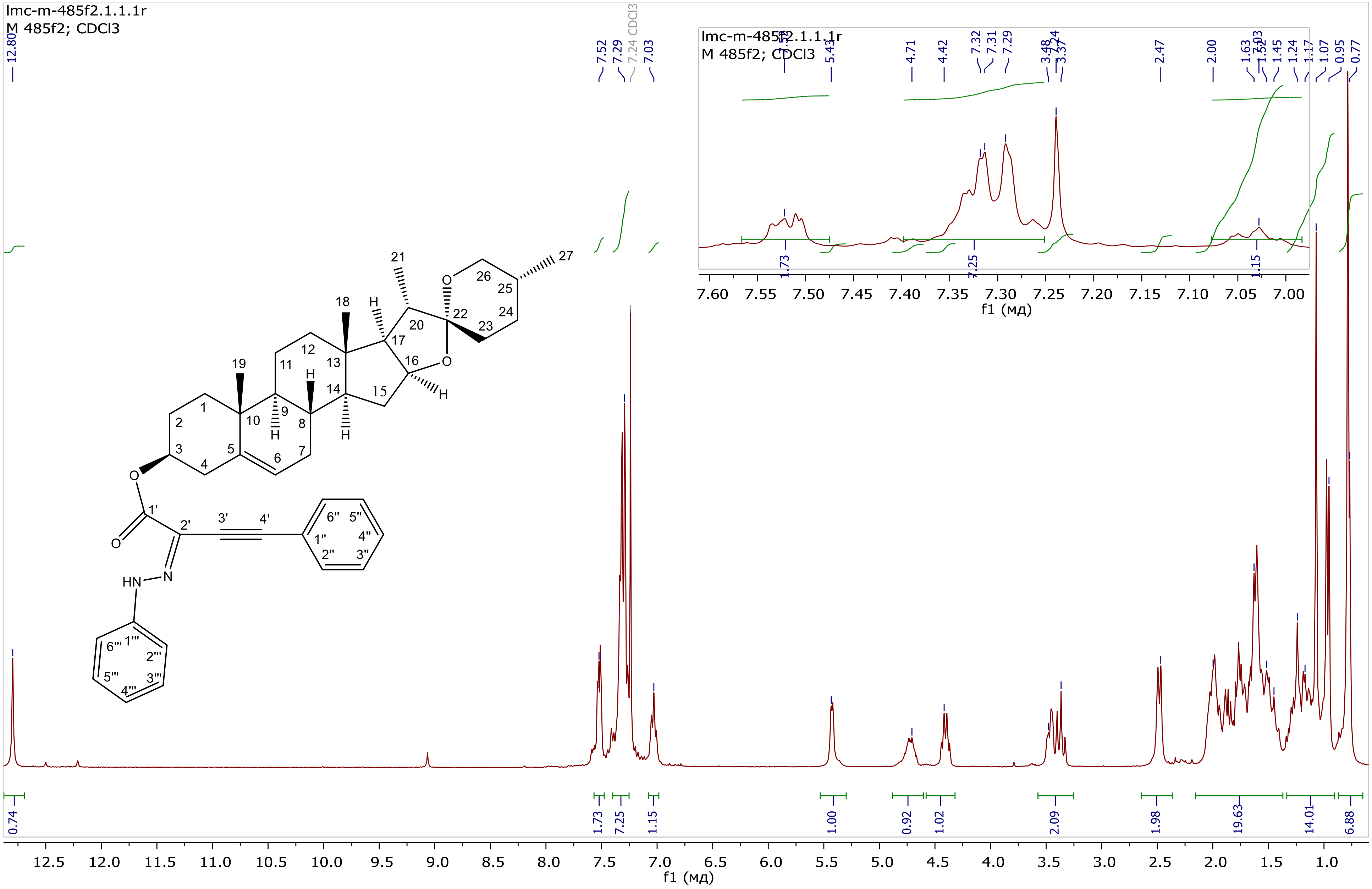

Compound 20 ((22*R*,25*R*)-spirost-5-en-3*β*-yl (*Z*)-4-phenyl-2-(2-phenylhydrazineylidene)but-3-ynoate), ^{13}C NMR, 126 MHz, $CDCl_3$

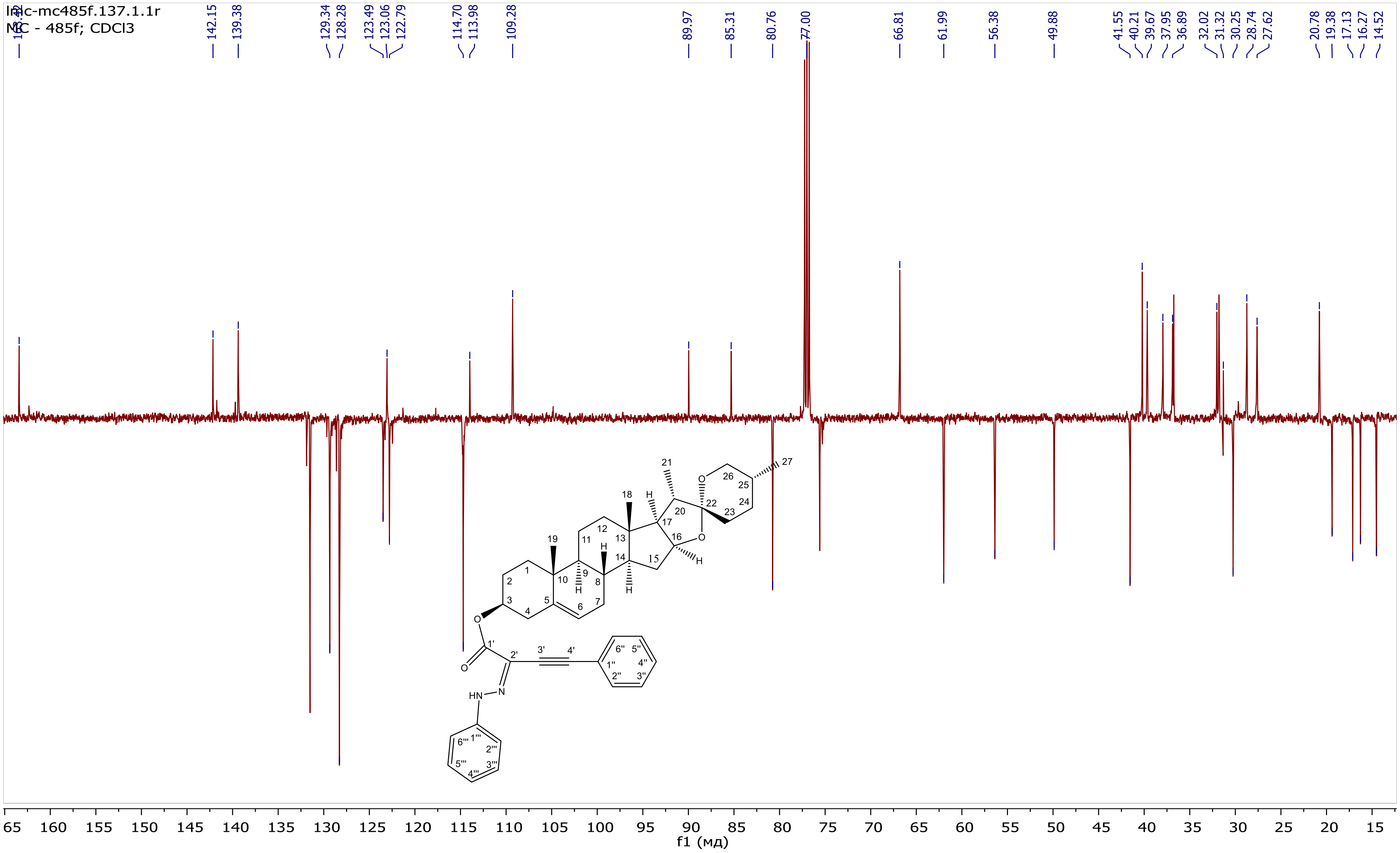

Compound 21 ((22*R*,25*R*)-spirost-5-en-3*β*-yl 1,3-diphenyl-1*H*-pyrazole-5-carboxylate), ^{1}H NMR, 600 MHz, $CDCl_3$

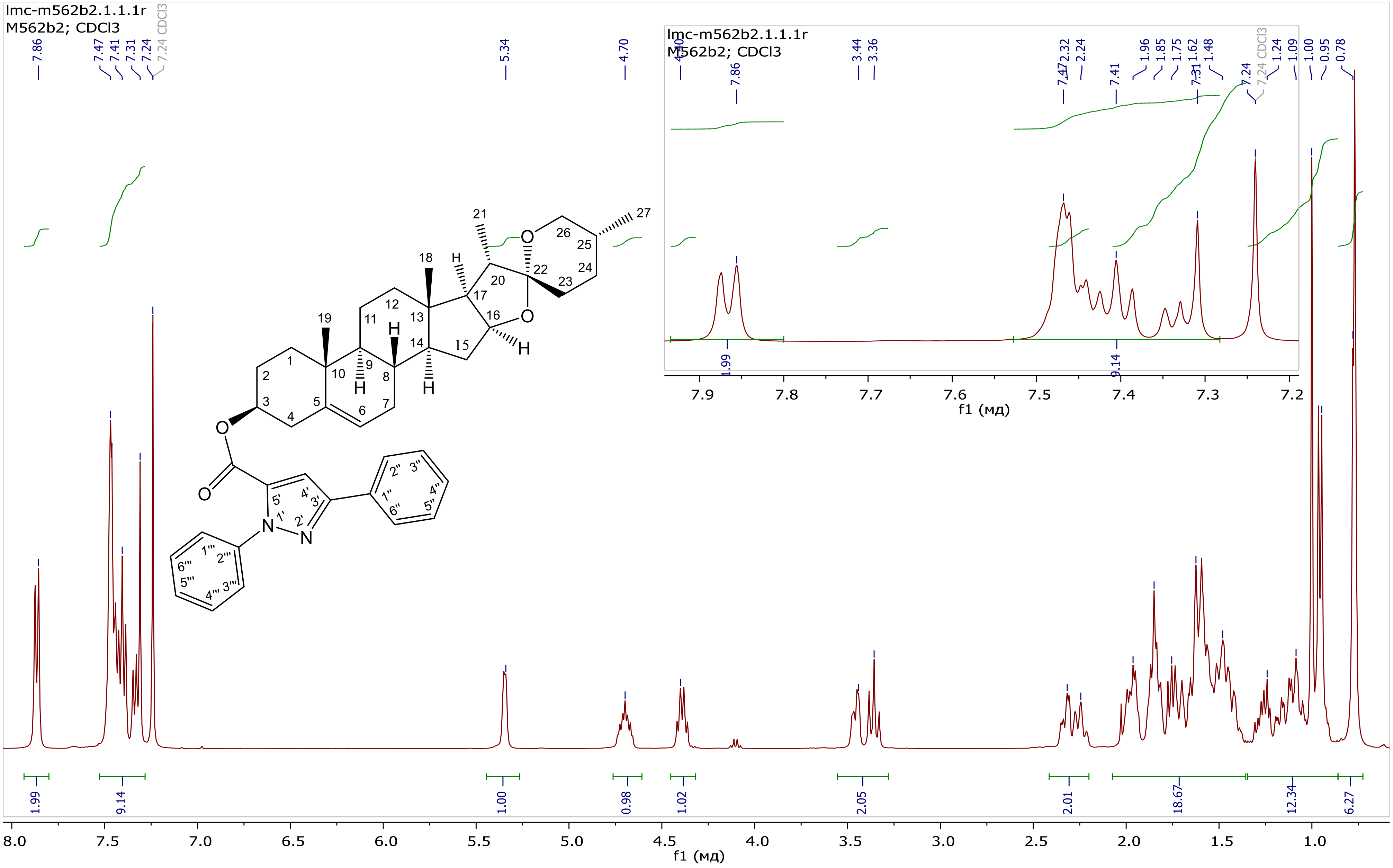

Compound 21 ((22*R*,25*R*)-spirost-5-en-3*β*-yl 1,3-diphenyl-1*H*-pyrazole-5-carboxylate), ^{13}C NMR, 126 MHz, $CDCl_3$

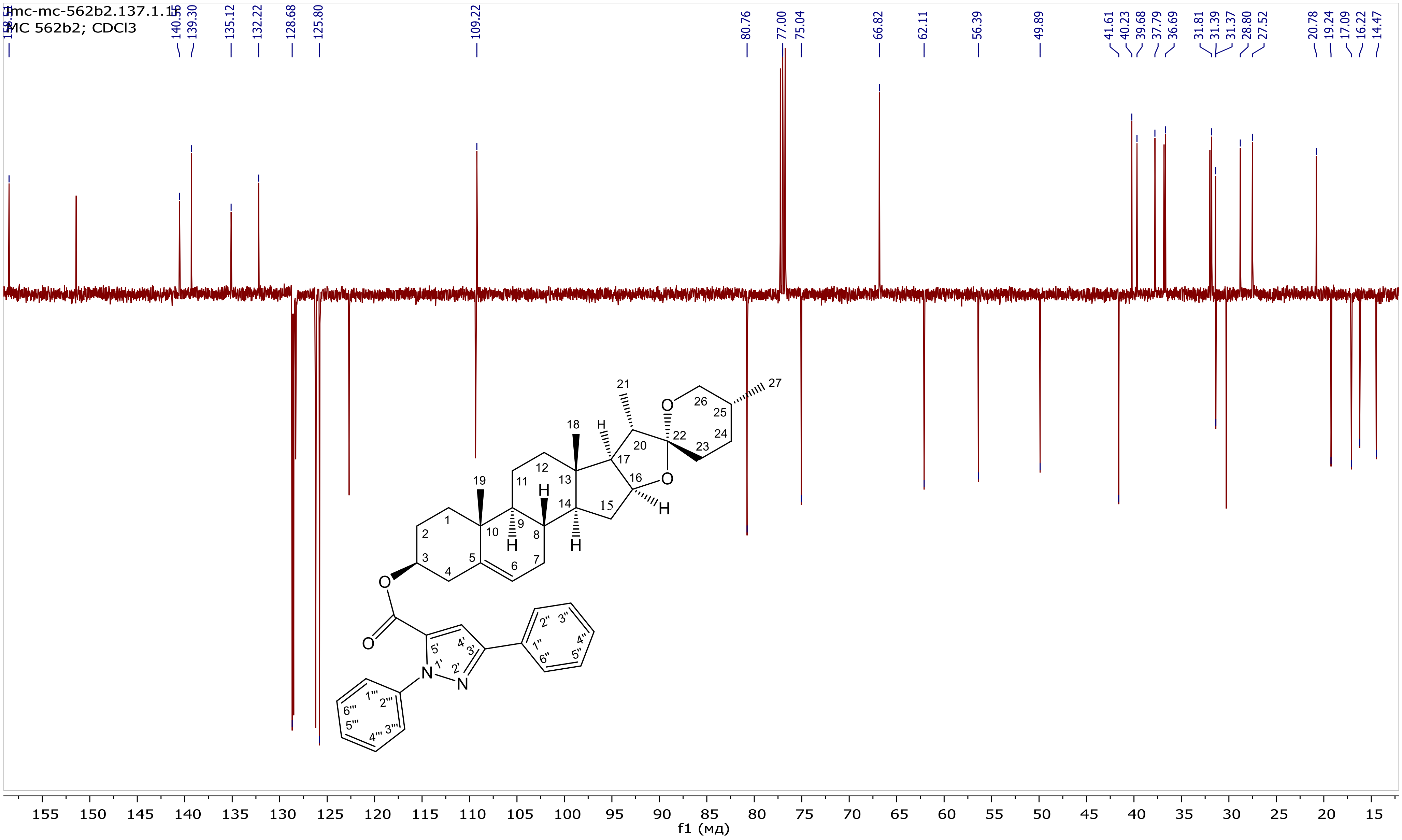

Compound 23 ((22*R*,25*R*)-spirost-5-en-3*β*-yl 3-phenyl-1*H*-pyrazole-5-carboxylate), ^{1}H NMR, 600 MHz, $CDCl_3$

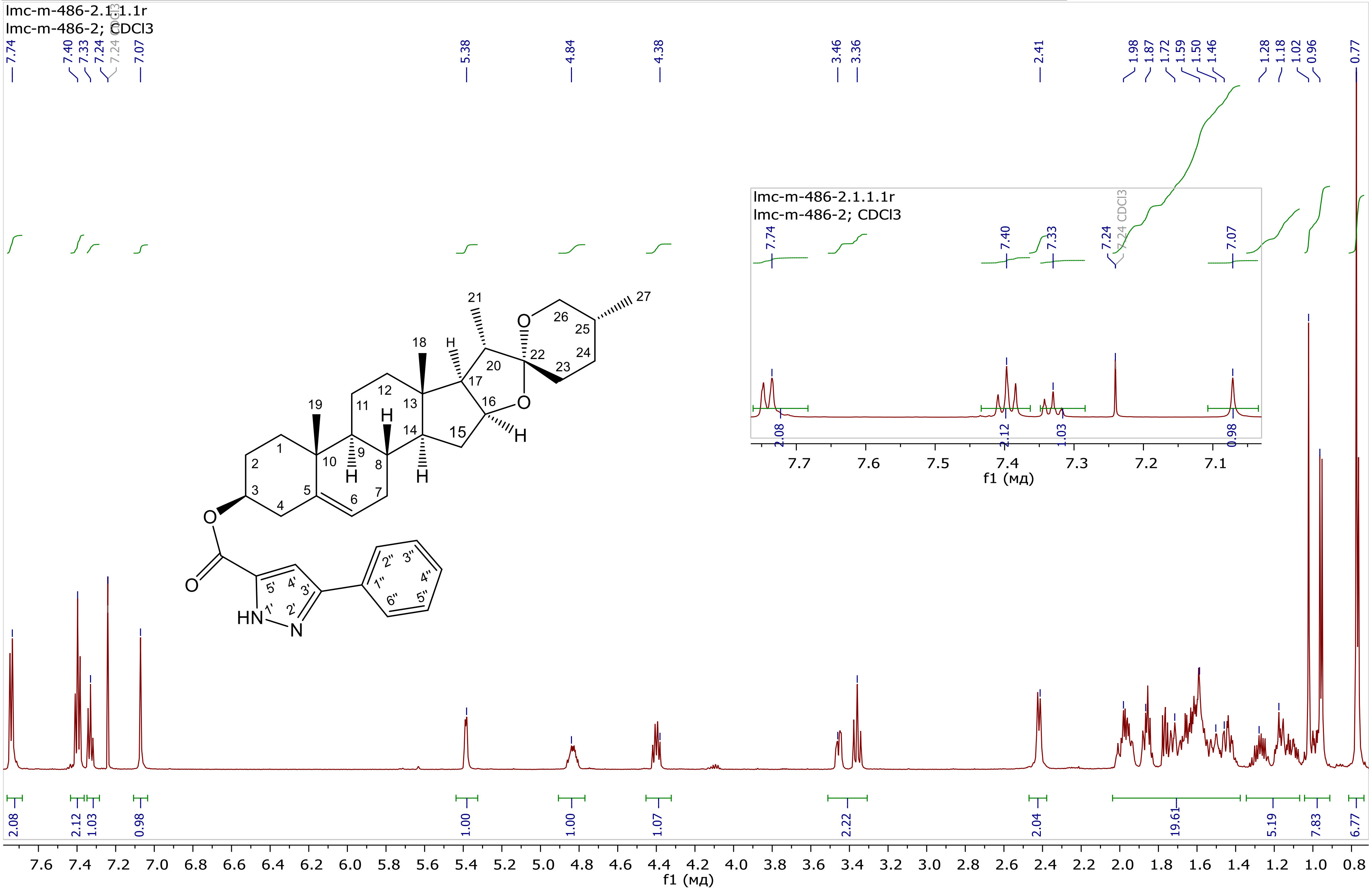

Compound 23 ((22*R*,25*R*)-spirost-5-en-3*β*-yl 3-phenyl-1*H*-pyrazole-5-carboxylate), ^{13}C NMR, 151 MHz, $CDCl_3$

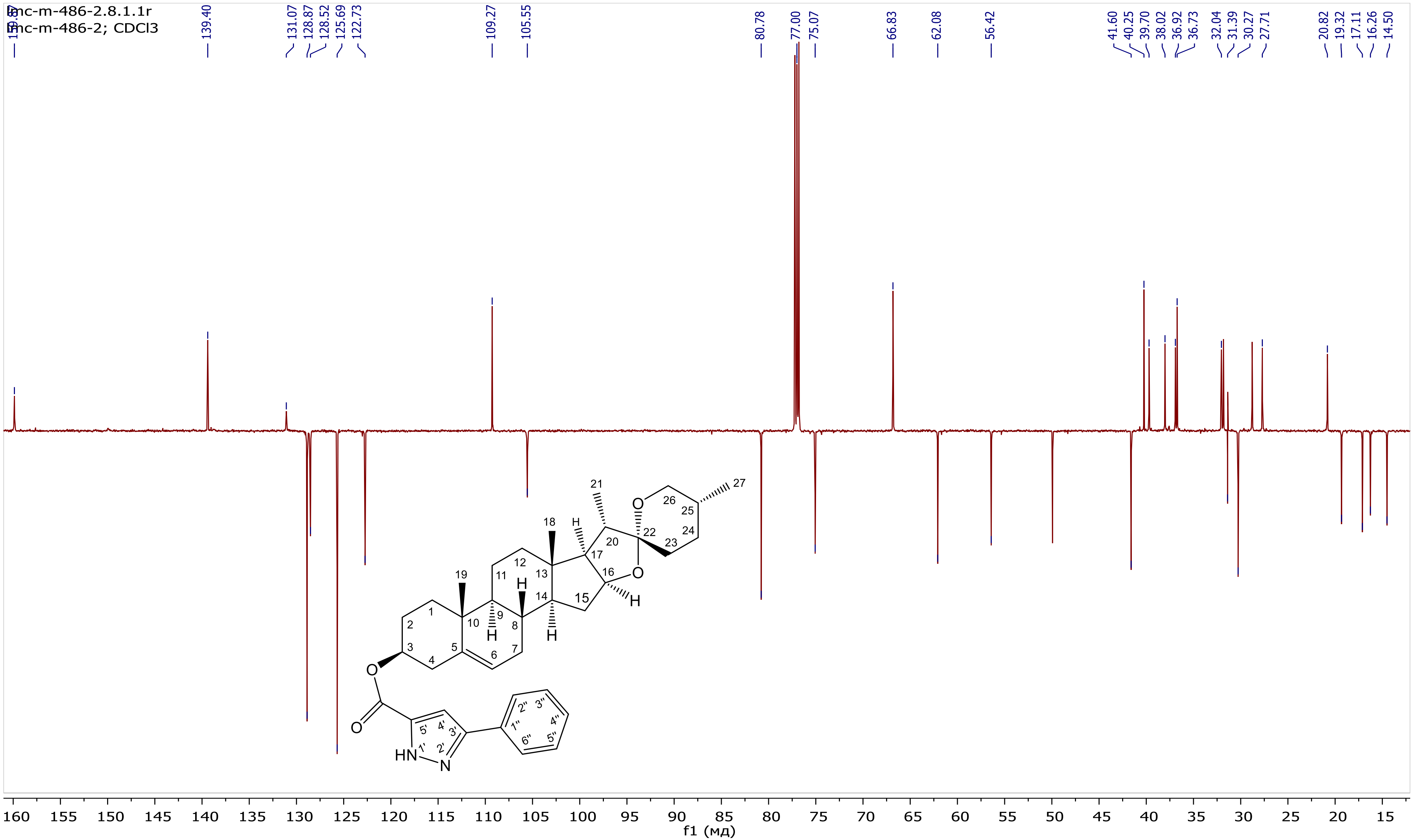

Compound 25 ((22*R*,25*R*)-spirost-5-en-3β-yl 2-benzoyl-3-hydroxy-5-phenyl-2,3-dihydro-1*H*-pyrazole-3-carboxylate), ^{1}H NMR, 600 MHz, $CDCl_3$

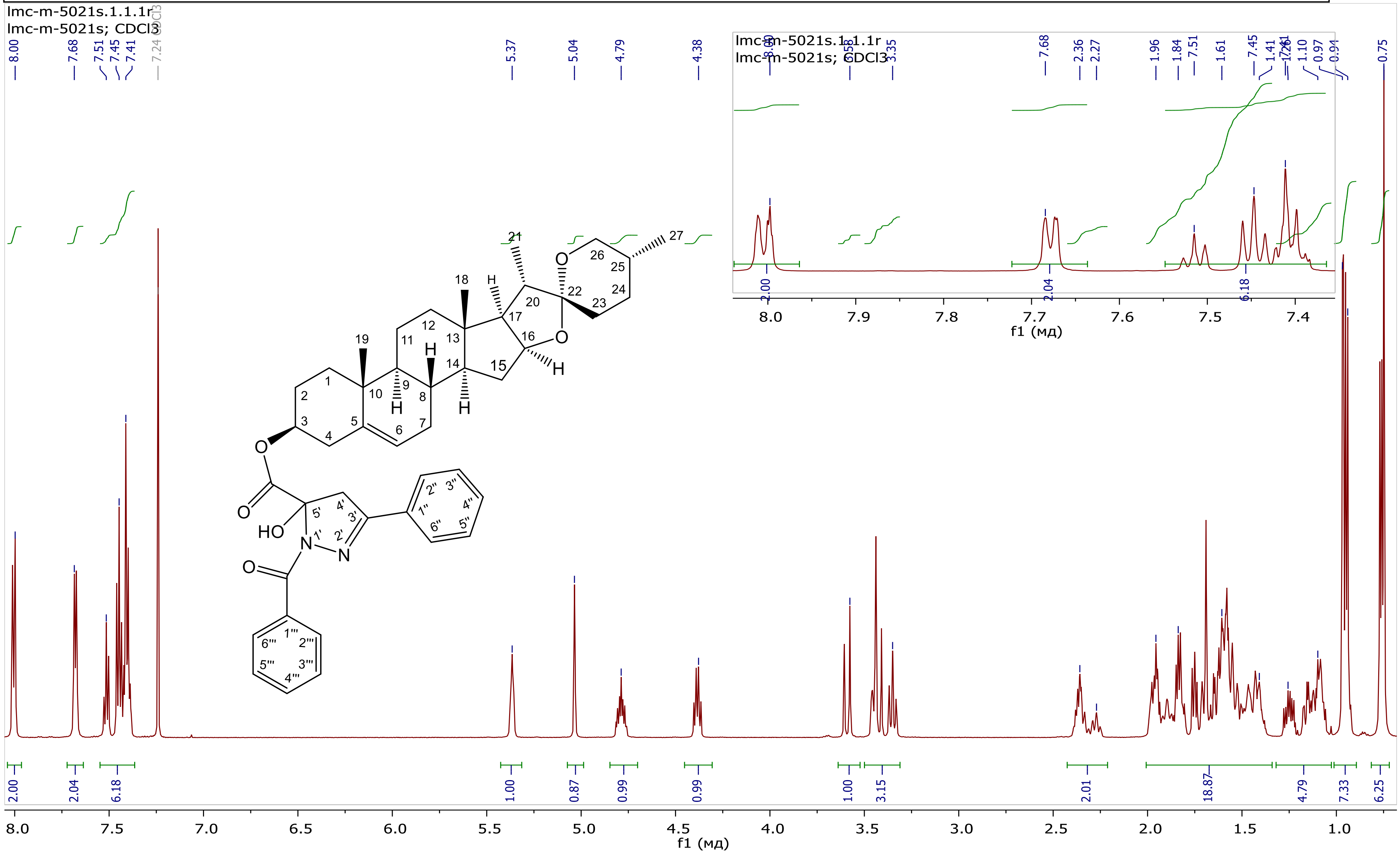

Compound 25 ((22*R*,25*R*)-spirost-5-en-3*β*-yl 2-benzoyl-3-hydroxy-5-phenyl-2,3-dihydro-1*H*-pyrazole-3-carboxylate), ^{13}C NMR, 151 MHz, $CDCl_3$

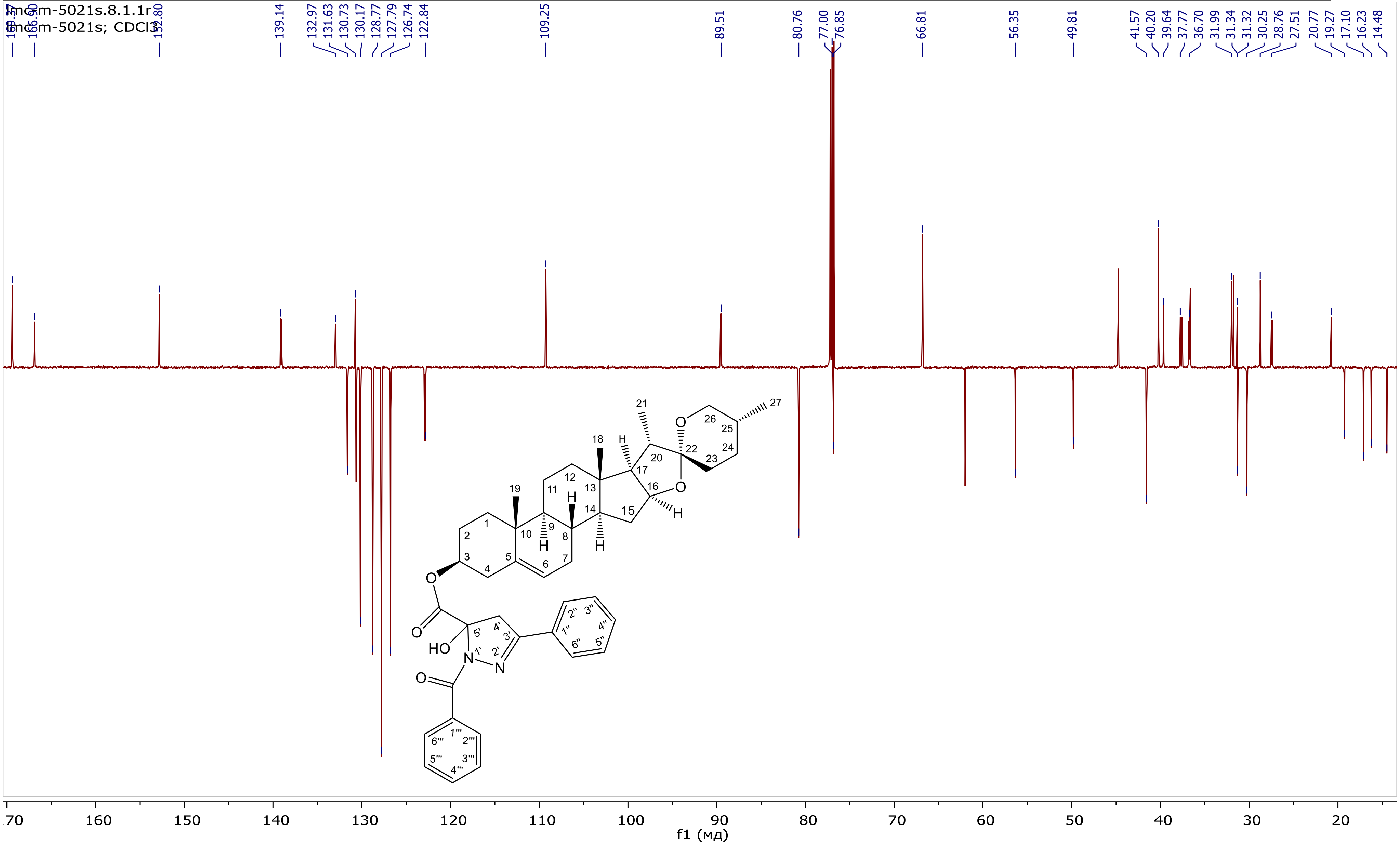

Compound 26 ((22*R*,25*R*)-spirost-5-en-3*β*-yl 1-(4-bromobenzoyl)-5-hydroxy-3-phenyl-4,5-dihydro-1*H*-pyrazole-5-carboxylate), ^{1}H NMR, 600 MHz, $CDCl_3$

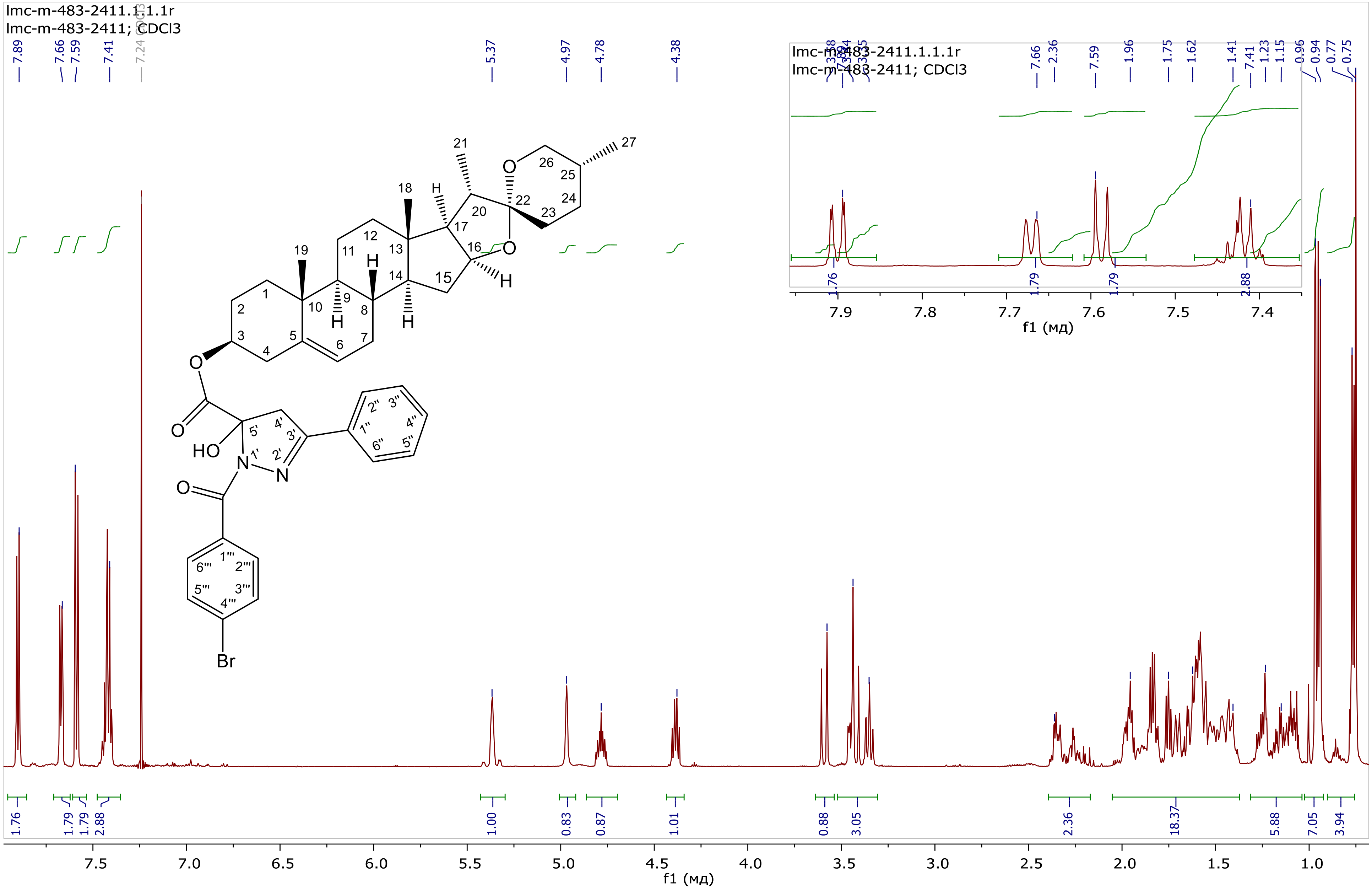

Compound 26 ((22*R*,25*R*)-spirost-5-en-3*β*-yl 1-(4-bromobenzoyl)-5-hydroxy-3-phenyl-4,5-dihydro-1*H*-pyrazole-5-carboxylate), ^{13}C NMR, 151 MHz, $CDCl_3$

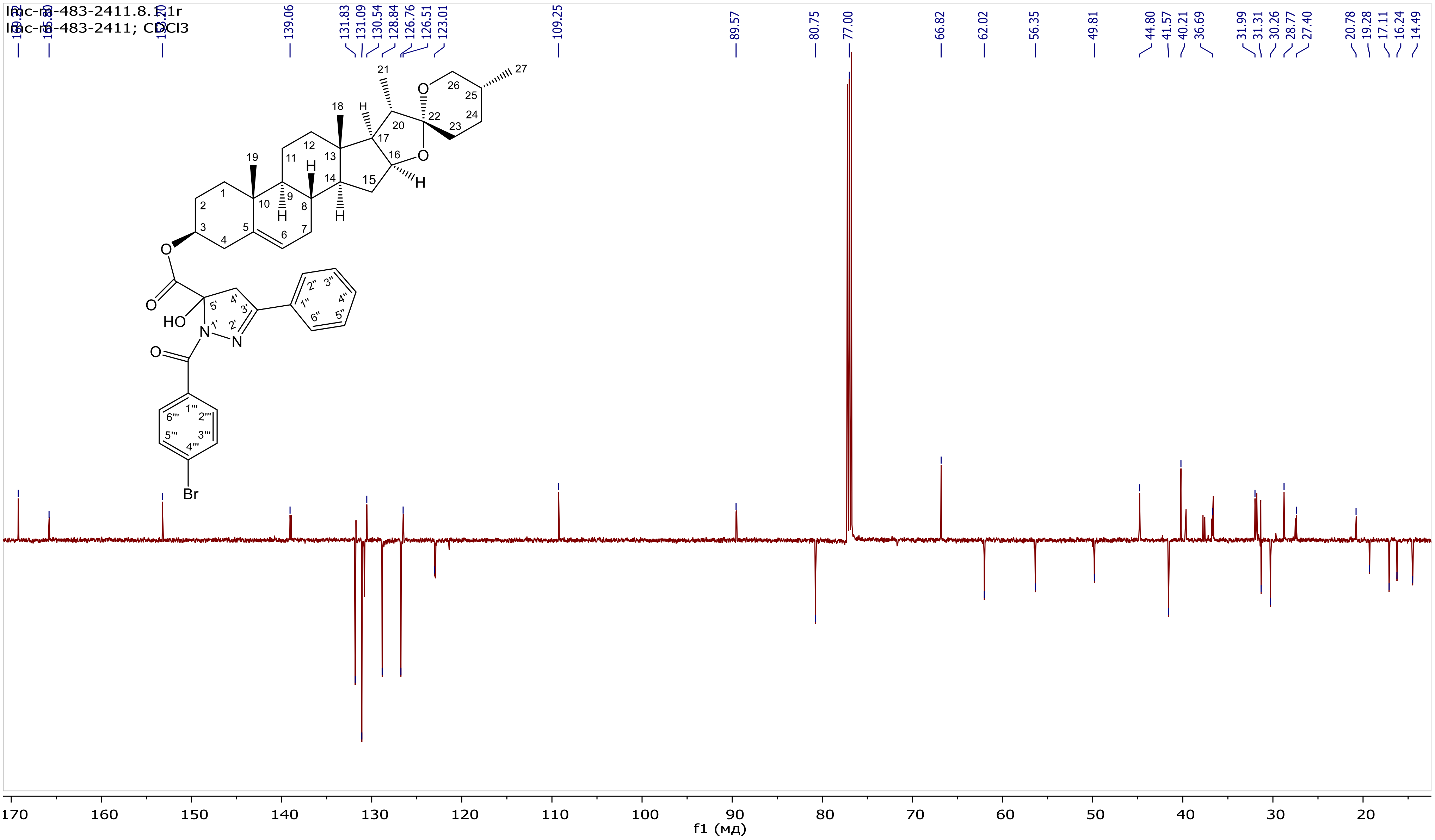

Supplement: Supplementary file 1 [file molecules-27-00162-s001.zip › molecules-1501631-supplementary.pdf]
